# Inducible deletion of skeletal muscle AMPKα reveals that AMPK is required for nucleotide balance but dispensable for muscle glucose uptake and fat oxidation during exercise

**DOI:** 10.1016/j.molmet.2020.101028

**Published:** 2020-06-03

**Authors:** Janne R. Hingst, Rasmus Kjøbsted, Jesper B. Birk, Nicolas O. Jørgensen, Magnus R. Larsen, Kohei Kido, Jeppe Kjærgaard Larsen, Sasha A.S. Kjeldsen, Joachim Fentz, Christian Frøsig, Stephanie Holm, Andreas M. Fritzen, Tine L. Dohlmann, Steen Larsen, Marc Foretz, Benoit Viollet, Peter Schjerling, Peter Overby, Jens F. Halling, Henriette Pilegaard, Ylva Hellsten, Jørgen F.P. Wojtaszewski

**Affiliations:** 1 Section of Molecular Physiology, Department of Nutrition, Exercise and Sports, Faculty of Science, University of Copenhagen, DK-2100, Copenhagen, Denmark; 2 Section of Systems Biology Research, Department of Biomedical Sciences, Faculty of Health, University of Copenhagen, Denmark; 3 Clinical Research Centre, Medical University of Bialystok, Bialystok, Poland; 4 Université de Paris, Institut Cochin, INSERM, CNRS, F-75014, Paris, France; 5 Institute of Sports Medicine Copenhagen, Department of Orthopedic Surgery M, Bispebjerg Hospital and Center for Healthy Aging, Faculty of Health and Medical Sciences, University of Copenhagen, Denmark; 6 Section for Cell Biology and Physiology, Department of Biology, University of Copenhagen, Denmark; 7 Section of Integrative Physiology, Department of Nutrition, Exercise and Sports, Faculty of Science, University of Copenhagen, DK-2100, Copenhagen, Denmark

**Keywords:** AMPK, Exercise, Glucose uptake, Muscle metabolism, Fat oxidation, Glycogen

## Abstract

**Objective:**

Evidence for AMP-activated protein kinase (AMPK)-mediated regulation of skeletal muscle metabolism during exercise is mainly based on transgenic mouse models with chronic (lifelong) disruption of AMPK function. Findings based on such models are potentially biased by secondary effects related to a chronic lack of AMPK function. To study the direct effect(s) of AMPK on muscle metabolism during exercise, we generated a new mouse model with inducible muscle-specific deletion of AMPKα catalytic subunits in adult mice.

**Methods:**

Tamoxifen-inducible and muscle-specific AMPKα1/α2 double KO mice (AMPKα imdKO) were generated by using the Cre/loxP system, with the Cre under the control of the human skeletal muscle actin (HSA) promoter.

**Results:**

During treadmill running at the same relative exercise intensity, AMPKα imdKO mice showed greater depletion of muscle ATP, which was associated with accumulation of the deamination product IMP. Muscle-specific deletion of AMPKα in adult mice promptly reduced maximal running speed and muscle glycogen content and was associated with reduced expression of UGP2, a key component of the glycogen synthesis pathway. Muscle mitochondrial respiration, whole-body substrate utilization, and muscle glucose uptake and fatty acid (FA) oxidation during muscle contractile activity remained unaffected by muscle-specific deletion of AMPKα subunits in adult mice.

**Conclusions:**

Inducible deletion of AMPKα subunits in adult mice reveals that AMPK is required for maintaining muscle ATP levels and nucleotide balance during exercise but is dispensable for regulating muscle glucose uptake, FA oxidation, and substrate utilization during exercise.

## Highlights

• Inducible deletion of AMPKα in adult mice disturbs nucleotide balance during exercise.
• Inducible deletion of AMPKα in adult mice lowers muscle glycogen content and reduces exercise capacity.
• Muscle mitochondrial respiration, and glucose uptake and FA oxidation during muscle contractions remain unaffected by AMPKα deletion.

## 1 Introduction

Physical activity is associated with a marked increase in muscle metabolism and energy turnover [1]. Therefore, maintaining intracellular levels of adenosine triphosphate (ATP) during exercise represents a metabolic challenge for the muscle cell. The increased ATP turnover during exercise leads to accumulation of intramyocellular adenosine monophosphate (AMP) in an exercise intensityand duration-dependent manner because of the adenylate kinase reaction (2ADP ↔ ATP + AMP) [2]. The increased intramyocellular AMP/ATP ratio leads to activation of the 5′-AMP-activated protein kinase (AMPK) [3], which promotes catabolic processes and inhibits anabolic processes to normalize the cellular energy status [4]. On this basis, skeletal muscle AMPK is proposed to function as a cellular energy sensor activated during exercise and thus to act as a central mediator of cellular signaling to maintain energy homeostasis.

Because of AMPK's pivotal role in the regulation of muscle metabolism, it provides a putative therapeutic target for metabolic disorders, such as type 2 diabetes (T2D) [5]. Acute pharmacological activation of AMPK in rodent muscle by different pharmacological agents (e.g., AICAR, PF739, MK-8722) promotes glucose disposal and fatty acid (FA) oxidation [[6], [7], [8]]. However, AMPK-deficient mouse models have provided conflicting results as to whether AMPK activation is required for muscle metabolism to cope with cellular energy stress during exercise. Although reduced muscle glucose uptake has been reported in AMPK-deficient mice during *in vivo* treadmill exercise and during contraction of isolated mouse muscles in some studies [[9], [10], [11], [12], [13], [14], [15]], other studies have demonstrated intact muscle glucose uptake during contractile activity [[16], [17], [18], [19], [20], [21], [22]]. Knockout (KO) of the two regulatory AMPKβ subunits (AMPKβ1β2M−KO) is associated with impaired muscle glucose uptake and increased FA oxidation during treadmill exercise [9], and KO of both catalytic AMPKα subunits (AMPKα1/α2) in muscle (AMPKα mdKO mice) or KO of the AMPK upstream kinase LKB1 (liver kinase B1) (LKB1 KO mice) leads to increased reliance on glucose as a substrate during treadmill exercise [16,17]. However, a direct interpretation of these findings is confounded by disrupted mitochondrial capacity [9,17] and changes in expression of key proteins/enzymes involved in lipid metabolism (e.g. CD36 and FABPpm) in these models [16,17]. In most studies, maximal treadmill running speed is reduced in AMPK-deficient mice compared to littermate controls (see [23] for detailed review). During high metabolic stress, the muscle cell prevents accumulation of AMP by converting it to inosine monophosphate (IMP) in an AMP deaminase (AMPD) dependent reaction that serves to maintain a homeostatic ATP/ADP ratio [24]. Accelerated ATP degradation and reduced glucose uptake have been observed in skeletal muscle of mice overexpressing a kinase-dead AMPKα2 construct (AMPKα2 KD mice) [10]. Whether these findings can be ascribed directly to the lack of functional AMPK or should be considered a consequence of the marked impairment in mitochondrial function reported for this model remains unclear [10].

In summary, the findings in the literature on AMPK-deficient mouse models indicate that the observed phenotypes may be ascribed to secondary effects due to the lifelong lack of AMPK rather than the acute regulation of AMPK activity. To study the direct effect(s) of AMPK activation during exercise, we developed a new mouse model where AMPK catalytic activity can be deleted in a muscle-specific manner at a specific time point in adult mice. With this new model, we attempted to clarify the direct role of AMPK in exercise-stimulated regulation of muscle metabolism.

## 2 Materials and methods

### 2.1 Generation of the tamoxifen-inducible muscle-specific AMPKα double knockout mouse model (AMPKα imdKO)

Inducible muscle-specific double AMPKα1/α2 KO mice (AMPKα imdKO) were generated by breeding double-floxed AMPKα1α2 mice (AMPKα1^fl/fl^, AMPKα2^fl/fl^) [15] with mice expressing a tamoxifen-inducible Cre-recombinase driven by the human skeletal actin promoter (HSA-MCM^+/-^) [25]. Deletion of AMPKα1/α2 in skeletal muscle was achieved by intraperitoneal injection of tamoxifen (Cat. No. T5648, Sigma–Aldrich) dissolved in 99% ethanol and resuspended in sunflower seed oil (Cat. No. S5007, Sigma–Aldrich). The tamoxifen treatment protocol comprised 3 single injections (40 mg/kg body weight) each separated by 48 h. Female double-floxed AMPKα1α2 control mice (AMPKα1^fl/fl^, AMPKα2^fl/fl^, HSA-MCM^−/−^) and AMPKα imdKO mice (AMPKα1^fl/fl^, AMPKα2^fl/fl^, HSA-MCM^+/-^) on a mixed background (C57/Bl6 ∼87.5% and SV129 ∼12.5%) were used in all experiments.

Initially, a time course study was performed to determine the earliest time point for optimal deletion of skeletal muscle AMPKα protein. For this time course experiment, mice were investigated 1, 3, and 8 weeks after the final tamoxifen injection and compared to vehicle-injected control mice (sunflower seed oil injections). Three weeks after the last tamoxifen injection was the earliest time point with optimal deletion of AMPKα protein; therefore, all subsequent experiments were performed ∼3 weeks after the final tamoxifen injection. Both the AMPKα imdKO mice and AMPKα double-floxed control littermates aged 12 ± 5 weeks (mean ± SD) were treated with tamoxifen. The tamoxifen administration protocol applied in this study resulted in substantial testicular swelling in male mice (unpublished observations); thus, for ethical and experimental reasons, we performed all subsequent experiments in female mice only. All mice had free access to water and rodent chow and were maintained on a 12:12 h light–dark cycle. All experiments were approved by the Danish Animal Experiments Inspectorate (License #2013-15-2934-00911, #2014-15-2934-01037 and #2019-15-0201-01659) and complied with the European Union guidelines for the protection of vertebra animals used for scientific purposes.

### 2.2 Body composition and morphological analyses

Body composition was measured before and ∼3 weeks after the final tamoxifen injection by the use of magnetic resonance imaging (EchoMRI 4-in-1; EchoMRI, Houston, TX). Skeletal muscle, heart, white adipose tissue, liver, and kidney were carefully dissected from anesthetized control and AMPKα imdKO mice and visually inspected for signs of disparity. Tissue mass was determined with 0.1 mg accuracy (ED124S, Sartorius, Goettingen, Germany).

### 2.3 Basal calorimetry

Before the measurements, mice were acclimatized for 3 days to individually housed airtight calorimetric cages connected to an indirect calorimetric system (Phenomaster/LabMaster system; TSE Systems, Bad Homburg, Germany). O_2_ consumption (VO_2_), CO_2_ production (VCO_2_), food intake, and physical activity level (laser beam breaks) were recorded for a 48-hour period while the mice were receiving a chow diet (Altromin no. 1324; Brogaarden, Horsholm, Denmark) and then a high-fat diet (HFD; 60% kcal derived from fat; no. D12492; Brogaarden, Horsholm, Denmark). After a 24-hour washout period on a regular chow diet, the effect of 24 h of fasting was investigated. For all calorimetric measurements, mice were maintained on a 12 h:12 h light–dark cycle and housed at 20–21 °C.

### 2.4 Treadmill acclimatization and maximal exercise capacity test

On 3 separate days (Pre, 1 week, and 3 weeks after final tamoxifen injection), mice were adapted to a treadmill running system (TSE Systems). The adaptation protocol comprised 5 min rest followed by 5 min running at 7.2 m/min and 5 min running at 9.6 m/min [16]. After 1 day of rest, a graded exercise capacity test was performed. The test was initiated by 5 min rest and the following task was treadmill running at 4.8 m/min where the treadmill speed increased 2.4 m/min every 2 min at a 5° incline. Mice were forced to run by the use of pressurized air and an electric shocker grid at the back of the treadmill. Exhaustion was reached when the mice stayed on the shocking grid despite repeated agitation with pressurized air. The last passed speed level was defined as maximal running speed.

### 2.5 Substrate utilization during treadmill exercise

Substrate utilization during treadmill exercise was investigated at least 48 h after the maximal exercise capacity test by measuring O_2_ consumption (VO_2_) and CO_2_ production (VCO_2_) in an airtight treadmill running system (CaloSys apparatus; TSE Systems, Germany). Mice were placed on the calorimetric treadmill and allowed to rest for 10 min. Next, the mice were forced to perform a 5-min warm-up at 40% of maximal treadmill running speed and 30 min continuous running at 60% of individual maximal running speed at a 5° incline. Measurements continued 15 min into exercise recovery.

### 2.6 Glucose uptake measurements during treadmill exercise

Mice either rested or exercised for 30 min at 70% of individual maximal running speed at a 15° incline. Intraperitoneal injection of saline containing [^3^H]-2-deoxyglucose ([^3^H]-2-DG) (8 ml/kg, 60 μCi/ml, Perkin Elmer, USA) was administered 20 min before the onset of exercise/rest. Blood samples in combination with blood glucose measurements (Contour XT, Bayer, Germany) were obtained from the tail vein immediately before and after exercise/rest to determine specific radioactivity in the blood. Mice were euthanized by cervical dislocation immediately after the last blood sample was drawn, and tissues were quickly harvested and frozen in liquid nitrogen. Muscle 2-DG uptake was determined as [^3^H]-2-deoxy-D-glucose-6-phosphate ([^3^H]-2-DG-6-P) content by Somogyi and perchloric acid precipitations of muscle homogenate, as described in the literature [16,26].

### 2.7 Glucose and insulin tolerance tests

For all tests, mice were individually housed. For the glucose tolerance test (GTT), mice were fasted for 5 h in the morning before they were given an intraperitoneal injection of glucose (2 g/kg body weight) dissolved in a 0.9% saline solution. For the insulin tolerance test (ITT), overnight fed mice were fasted for 2 h in the morning before they were given an intraperitoneal injection of insulin (1 U/kg body weight, Actrapid, Novo Nordisk, Bagsværd, Denmark). Blood was collected from the tail vein at 0, 20, 40, 60, 90 and 120 min in the GTT and 0, 20, 40 and 60 min in the ITT. Blood glucose concentrations were determined by using a glucometer (Contour XT, Bayer, Germany). For the GTT, plasma insulin concentrations were determined at 0, 20, and 40 min by using an enzyme-linked immunosorbent ELISA assay (Cat. No. 80-INSMSU-E10, ALPCO) according to the manufacturer's instructions.

### 2.8 Insulin-stimulated and contraction-stimulated glucose uptake in isolated muscles

Fed mice were anesthetized by an intraperitoneal injection of pentobarbital (10 mg/100 g body weight) and xylocain (0.5 mg/100 g body weight) before the soleus and extensor digitorum longus (EDL) muscles were excised and suspended at resting tension in incubation chambers (model 610/820M, DMT, Denmark) containing Krebs–Ringer buffer (KRB; 117 mM NaCl, 4.7 mM KCl, 2.5 mM CaCl_2_, 1.2 mM KH_2_PO_4_, 1.2 mM MgSO_4_, 0.5 mM NaHCO_3_, pH 7.4) supplemented with 0.1% bovine serum albumin (BSA), 2 mM Na-pyruvate, and 8 mM Mannitol. During the entire incubation period, the buffer was maintained at 30 °C and continuously gassed with carbogen (95% O_2_ and 5% CO_2_). After ∼30 min preincubation, basal, submaximal (100 μU/ml) and maximal (10,000 μU/ml) insulin-stimulated muscle glucose uptake was determined during the last 10 min of a 30 min stimulation period by adding 1 mM [^3^H]-2-DG (0.028 MBq/ml) and 7 mM [^14^C]-Mannitol (0.0083 MBq/ml) to the incubation medium. For contraction-stimulated glucose uptake, the muscles were electrically stimulated to contract (1 s train/15 s, 0.2 ms pulses, 100 Hz, 30 V; MultiStim System-D330, Harvard Apparatus) for 10 min. 2-DG uptake during contraction was measured by adding 1 mM [^3^H]-2-DG (0.028 MBq/ml) and 7 mM [^14^C]-Mannitol (0.0083 MBq/ml) to the incubation medium immediately before initiation of muscle contraction. After incubation, the muscles were harvested, washed in ice-cold saline, blotted dry, and quickly frozen in liquid nitrogen. Uptake of 2-DG was determined as described in the literature [27].

### 2.9 Contraction-stimulated FA oxidation in isolated soleus muscles

Contraction-stimulated exogenous palmitate oxidation in isolated soleus muscle was measured in a manner similar to that in the literature [17]. In summary, excised soleus muscles from anesthetized mice were mounted at resting tension (∼5 mN) in vertical incubation chambers (Radnoti, Monrovia, CA) containing 30 °C carbogenated (95% O_2_ and 5% CO_2_) KRB supplemented with 5 mM glucose, 2% fat-free BSA, and 0.5 mM palmitate. After ∼20 min of preincubation, the incubation buffer was replaced with KRB additionally containing [1–^14^C]-palmitate (0.0044 MBq/ml). To seal the incubation chambers, mineral oil (Cat. No. M5904, Sigma–Aldrich) was added on top. Exogenous palmitate oxidation was measured at rest and during 25 min contractions (18 trains/min, 0.6 s pulses, 30 Hz, 60 V). After incubation, incubation buffer and muscles were collected to determine the rate of palmitate oxidation as previously described [17,28]. Palmitate oxidation was determined as CO_2_ production (complete FA oxidation) and acid-soluble metabolites (representing incomplete FA oxidation). As no difference was observed in complete and incomplete FA oxidation between genotypes, palmitate oxidation is presented as a sum of these 2 forms.

### 2.10 Mitochondrial respiration of permeabilized skeletal muscle fibers

After excision, tibialis anterior (TA) muscles were immediately transferred to ice-cold BIOPS buffer (10 mM Ca-EGTA, 0.1 μM free calcium, 20 mM imidazole, 20 mM taurine, 50 mM K-MES, 0.5 mM DTT, 6.56 mM MgCl_2_, 5.77 mM ATP, 15 mM phosphocreatine, pH 7.1). Adipose and connective tissue were removed, and muscle fibers were mechanically separated into small fiber bundles (∼3 mg) with fine forceps to maximize surface area and minimize diffusion limitations. Permeabilization of fiber bundles was performed by a 30-min saponin treatment (30 μg/mL in BIOPS) on a rotator at 4 °C. After permeabilization, fiber bundles were washed for at least 30 min in MiR05 buffer (0.5 mM EGTA, 3 mM MgCl_2_, 60 mM K-lactobionate, 20 mM taurine, 10 mM KH_2_PO_4_, 20 mM Hepes, 110 mM sucrose, 1 g/L BSA, pH 7.1) before analyses. Mitochondrial respiration was measured in duplicates in permeabilized muscle fiber bundles under hyperoxic conditions ([O_2_] ∼400-200 μM) at 37 °C in MiR05 medium by using the Oxygraph-2k (Oroboros Instruments, Innsbruck, Austria). Complex I supported leak respiration was measured after the addition of 5 mM pyruvate, 10 mM glutamate, and 2 mM malate. Maximal complex I supported oxidative phosphorylation (OXPHOS) capacity (CI_*P*_) was measured after the addition of ADP (4 mM). Complex I+II supported OXPHOS capacity (CI+II_*P*_) was measured after the addition of succinate (10 mM). Electron transfer system (ETS) capacity through complex I+II was measured after sequential addition of 0.5 μM FCCP. Finally, ETS capacity through complex II (CII) was achieved by adding 1 μM rotenone to inhibit complex I. After each respiration protocol, permeabilized fiber bundles were extracted from the respiration chamber and weighed after vacuum drying, and data are expressed as oxygen flux relative to muscle dry weight.

### 2.11 Muscle glycogen, nucleotide, AMPD activity and lactate measurements

Muscle glycogen content was determined by a fluorometric method as glycosyl units after acid hydrolysis of 10–15 mg wet weight muscle samples [29]. Muscle specimens from quadriceps muscle were extracted in perchloric acid and analyzed for nucleotides by reverse-phase HPLC. AMPD activity was measured in quadriceps muscle by adding 2 μl muscle homogenate to 1000 μl of the reagent solution containing 12.5 mM AMP and the formation of IMP was analyzed by HPLC. Kinetic properties for AMPD (V_max_ and K_m_) were analyzed in muscle homogenate in the presence of 15 mM, 0.1 mM, 0.06 mM, and 0.04 mM AMP, and the formation of IMP was quantified by HPLC as reported in the literature [30]. Muscle lactate concentration was determined in crushed quadriceps muscle by a fluorometric method previously described [29].

### 2.12 mRNA isolation, reverse transcription and real-time PCR

A whole EDL muscle (∼10 mg) and ∼25 mg of quadriceps muscle were crushed in liquid nitrogen and subsequently homogenized before RNA was isolated by the guanidine thiocyanate phenol-chloroform method [31]. The pellet was washed twice in 75% EtOH (−20 °C) and centrifuged for 5 min at 12,000 g and 4 °C between washes. The pellet was vacuum dried after last wash and resuspended in 1 μl/(mg initial muscle) 0.1 mM EDTA. RNA concentration was determined by using a Nanodrop1000 spectrophotometer (Thermo Fischer Scientific, Waltham, USA) and RNA purity was determined by the 260/280 nm ratio. Reverse transcriptase reaction was performed on 3 μg of total RNA by using the Superscript II RNase H- system (ThermoFisher Scientific, Waltham, USA) as previously described [32]. The mRNA content of specific genes was determined by fluorescence-based real-time PCR (ABI PRISM 7900 Sequence Detection System, Applied Biosystems). The forward and reverse primers and TaqMan probes were either designed from mouse-specific sequence data (Entrez-NIH and Ensembl, Sanger Institute) by using computer software (Primer Express, Applied Biosystems) or purchased as kits from ThermoFisher Scientific. PCR amplification was performed in triplicates of 10 μl with 10 ng of cDNA as described in the literature [33] and with TATA-Box Binding Protein (TBP) as the endogenous control. TBP has been described as an endogenous reference gene [34], and we found that TBP was unaffected by the intervention and genotype in this study. A detailed list of primer and probe sequences is provided in supplemental 1. The cycle threshold (Ct) values of the unknown samples were converted to an amount by using a relative standard curve derived from a dilution series of a representative pool. For each sample, the amount of the specific mRNA analyzed was then divided by the mRNA amount of the housekeeping gene (TBP) for assay normalization.

### 2.13 Muscle processing

Muscles were homogenized in ice-cold buffer (10% glycerol, 20 mM Na-pyrophosphate, 150 mM NaCl, 50 mM Hepes 1% NP-40, 20 mM β-glycerophosphate, 10 mM NaF, 2 mM PMSF, 1 mM EDTA, 1 mM EGTA, 10 μg/ml aprotinin, 10 μg/ml leupeptin, 2 mM Na_3_VO_4_, and 3 mM benzamidine, pH 7.5) by using a TissueLyser II (QIAGEN, Hilden, Germany). Subsequently, homogenates were rotated end-over-end at 4 °C for 1 h. Muscle lysate was obtained as supernatant from homogenate by centrifugation for 20 min at 16,000 g and 4 °C. Protein abundance in muscle homogenates and lysates was determined in triplicate by the bicinchoninic acid method with BSA as protein standards (Thermo Fisher Scientific, Waltham, USA).

### 2.14 Glycogen synthase (GS)-activity

GS-activity in muscle homogenates was measured in 96-well microtiter assay plates (Unifilter 350 plates; Whatman, Cambridge, UK) as described in the literature [35]. GS-activity was reported as a percentage of fractional velocity (% FV) and calculated as 100 x activity in the presence of 0.17 mM glucose-6-phosphate (G6P) divided by activity in the presence of 8 mM G6P (saturated).

### 2.15 SDS-PAGE and Western blotting

Muscle lysates were prepared in sample buffer and heated for 5 min at 96 °C. Equal amounts of protein were loaded on self-cast gels and separated by SDS-PAGE. Gels were transferred to polyvinylidene fluoride membranes (Merck, Darmstadt, Germany) by using semidry blotting. Membranes were incubated for 5 min in TBST containing either 2% skim milk or 3% BSA and subsequently incubated overnight at 4 °C with primary antibody. A detailed list of antibodies is in the supplemental material (Supplemental 2). Proteins with bound primary and secondary antibodies were visualized by chemi-luminescence and a digital imaging system (ChemiDoc MP System, BioRad, California, USA). Linearity was assessed for all proteins to ensure that the obtained band intensity was within the dynamic range.

### 2.16 Statistical analyses

Data are presented as means ± SEM unless stated otherwise. Differences between AMPKα control and imdKO mice were analyzed by Students t-test or 2-way ANOVA with or without repeated measurements as appropriate and specified in the figure legends. The Student–Newman–Keuls test was used for post hoc testing. Statistical analyses were performed in Sigmaplot (version 13.0; SYSTAT, Erkrath, German), and P ≤ 0.05 was used as the significance level.

## 3 Results

### 3.1 Time course of skeletal muscle-specific deletion of AMPKα1 and α2

**Figure 1:**
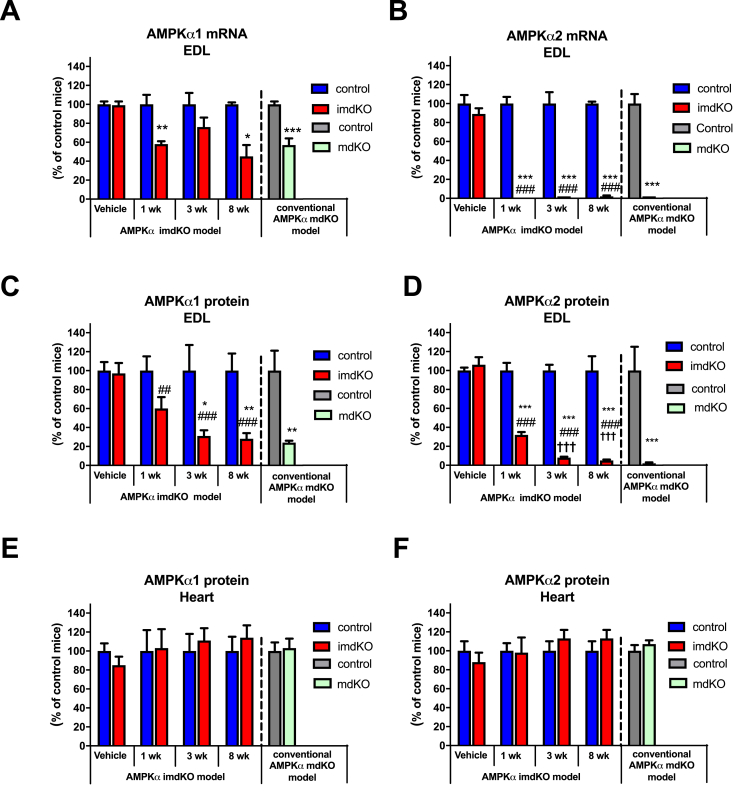
**Tamoxifen-induced deletion of AMPKα subunits in adult mice.** A–F: Muscle-specific deletion of AMPKα1 and α2 was obtained by expressing a tamoxifen-inducible Cre-recombinase construct driven by the human skeletal muscle actin promotor. The tamoxifen treatment protocol comprised 3 single injections (40 mg/kg bw) separated by 48 h, and mice were investigated 1, 3, and 8 weeks after the last tamoxifen injection. For the vehicle experiment, all mice received injections of sunflower oil. Gene expression of AMPKα1 and α2 subunits was measured in EDL muscle. Protein levels of AMPKα1 and α2 were measured in EDL and heart from control and AMPKα imdKO mice. These data from the AMPKα imdKO model were compared to the conventional AMPKα double KO model (AMPKα mdKO) with chronic lack of AMPK function. Protein levels were measured by immunoblotting, and gene expression was measured by real time PCR and presented relative to TATA-Box Binding Protein (TBP). Data have are normalized to control mice (=100%). Data are given as means ± SEM (n = 5–6 within each group). One-way ANOVA was used for comparing 1, 3, and 8 weeks to vehicle control within AMPKα imdKO mice. An additional t-test was applied to compare AMPKα imdKO with control mice within each time point. ∗p ≤ 0.05, ∗∗p ≤ 0.01, and, ∗∗∗p ≤ 0.001 for difference from corresponding control mice. ##p ≤ 0.01 and ###p ≤ 0.001 for difference from corresponding AMPKα imdKO vehicle. †††p ≤ 0.001 for difference from 1 week AMPKα imdKO.

To define the earliest time point at which full deletion of the catalytic AMPKα1 and α2 protein had occurred in myofibers, we investigated the AMPK subunit levels 1, 3, and 8 weeks after tamoxifen-induced gene deletion. One week after the last tamoxifen injection, the mRNA content of AMPKα1 and AMPKα2 in EDL muscle was reduced to ∼58% and ∼0%, respectively, compared to tamoxifen-treated AMPKα double-floxed control mice (Figure 1A, B). These changes in AMPKα gene expression were also present at 8 weeks and resulted in a marked reduction in AMPKα1 and near complete deletion of AMPKα2 protein content (Figure 1C–D and Supplementary Fig S3F). Thus, the AMPKα1 protein level was reduced to ∼60% in AMPKα imdKO compared to control EDL muscle 1 week after tamoxifen treatment and decreased further to ∼30% at 3 weeks after tamoxifen treatment ended (Figure 1C). The protein content of AMPKα2 in EDL muscle from AMPKα imdKO was reduced to ∼30 and ∼8% at 1 and 3 weeks after tamoxifen treatment, respectively (Figure 1D).

A similar pattern for tamoxifen-induced reduction in AMPKα1 and AMPKα2 protein levels was also observed in quadriceps muscle (Supplementary Fig S3A–B). Notably, protein levels of AMPKα1 and AMPKα2 in skeletal muscle from AMPKα imdKO mice measured 3 weeks after tamoxifen treatment ended corresponded to levels observed in the previously described conventional (chronic) AMPKα muscle-specific double knockout (mdKO) mouse model [16] (right bar in Figure 1C–D). The remaining amount of AMPKα1 protein in whole muscle samples from AMPKα imdKO mice is also found in the conventional AMPKα mdKO model and probably is derived from the non-muscle cells (e.g., blood cells, adipocytes, endothelial cells) in the crude muscle sample preparations. We expected the expression in non-muscle cells would be unaffected because of the skeletal muscle specificity of the HSA promotor [16]. AMPKα1 and AMPKα2 protein levels in the heart muscle remained similar between genotypes after tamoxifen treatment (Figure 1E–F and Supplementary Fig S3G), verifying that KO of the catalytic AMPK subunits indeed is specific for skeletal muscle myofibers. The AMPKβ2-associated heterotrimer complexes account for ∼95% of the total AMPK pool in mouse EDL muscle (α2β2γ1 ∼70%, α2β2γ3 ∼20% and α1β2γ1 ∼5%) [36], and in accordance, the marked reduction in AMPKα muscle protein levels observed in the present model is accompanied by a corresponding reduction in protein levels of the regulatory AMPK subunits β2 and γ1, and AMPK γ3 tended to be reduced (Supplementary Figs S3C–F). In absolute values, these protein levels resemble those in skeletal muscle of the conventional AMPKα mdKO mouse model.

Collectively, these data suggest that a maximal reduction in AMPKα1 and AMPKα2 protein levels is observed at the earliest 3 weeks after tamoxifen treatment ended. Based on these observations, our chosen time point for all subsequent experiments was 3 weeks.

### 3.2 Body composition and resting metabolism remain unaffected by inducible KO of AMPKα1 and α2 in skeletal muscle

Body composition of control and AMPKα imdKO mice was investigated before and 3 weeks after ended tamoxifen treatment by using magnetic resonance scanning (Table 1). Before tamoxifen treatment, no differences were observed between genotypes, both had normal growth rates and were born with the expected mendelian ratio (data not shown). A decrease in body weight (∼3%) and an increase in fat mass (∼10%) were observed in both genotypes 3 weeks after tamoxifen relative to before tamoxifen treatment. A minor decrease in lean body mass (∼3%) was observed in AMPKα imdKO but not in control mice after tamoxifen treatment. No significant difference between genotypes in lean body mass was evident before and after tamoxifen treatment. Careful dissection and weighing of individual organs revealed a similar weight and appearance of muscle, heart, subcutaneous adipose tissue, liver, and kidney from AMPKα imdKO and control littermates (Table 2). Resting metabolism during the light and dark periods on the chow diet was comparable between control and AMPKα imdKO mice (Supplementary Figs S4A–B). We also investigated the ability to switch substrate utilization toward FA oxidation, by subjecting the mice to 2 days of an HFD and a 24-hour fasting regimen. The HFD intervention lowered RER from ∼0.95 to ∼0.80 in both genotypes during the light period, and RER increased similarly in the 2 genotypes during the dark period (RER from ∼0.80 to ∼0.85; Supplementary Figs S4A and C). Compared to the chow diet, the 24-hour fasting intervention lowered RER markedly in the light period, and RER declined further during the dark period (RER ∼0.73 to ∼0.71 in both genotypes) with no difference between genotypes (Supplementary Figs S4D–E). Together, these data suggest that resting metabolism under these experimental conditions is similarly covered by FA oxidation in AMPKα imdKO and control mice. Oxygen consumption, spontaneous physical activity, and food intake during the diet interventions showed no difference between AMPKα imdKO and control littermates (Supplementary Figs S4F–P).

**Table 1:** Body composition of control and AMPKα imdKO mice.

	Pretamoxifen treatment		3 weeks post tamoxifen treatment		Main effect	Interaction
	control	imdKO	control	imdKO		
**Weight (g)**	26.3 ± 0.4	26.3 ± 0.5	25.7 ± 0.5	25.3 ± 0.4	∗∗∗	–
**Fat mass (g)**	2.0 ± 0.2	1.8 ± 0.2	2.2 ± 0.1	2.1 ± 0.1	∗	–
**LBM (g)**	21.9 ± 0.3	22.3 ± 0.4	21.8 ± 0.4	21.5 ± 0.3	–	p = 0.003

Body composition was investigated before (pre) and 3 weeks after (post) tamoxifen treatment. Data are given as means ± SEM (n = 20). ∗p < 0.05 and ∗∗∗p < 0.001 for significant difference from pretreatment independently of genotype.

**Table 2:** Tissue weight in control and AMPKα imdKO mice.

Tissue	Weight (mg)		p-value
	control	imdKO	
Tibialis anterior	42 ± 1	43 ± 1	0.49
Heart	101 ± 3	98 ± 3	0.52
WAT	200 ± 14	245 ± 37	0.28
Liver	991 ± 79	988 ± 78	0.98
Kidney	134 ± 6	136 ± 10	0.90

Weight (mg) of tibialis anterior muscle, heart, white subcutaneous adipose tissue (WAT), liver, and kidney was investigated 3 weeks after tamoxifen treatment. Data are given as means ± SEM (n = 8–12).

Collectively, these observations demonstrate that body composition, resting metabolism, and metabolic flexibility, that is, the ability to adjust metabolism according to different feeding and fasting regimes, remain unaffected by inducible deletion of AMPKα subunits in skeletal muscle of adult mice.

### 3.3 Normal whole-body insulin action and insulin-stimulated glucose uptake in isolated skeletal muscle from AMPKα imdKO mice

**Figure 2:**
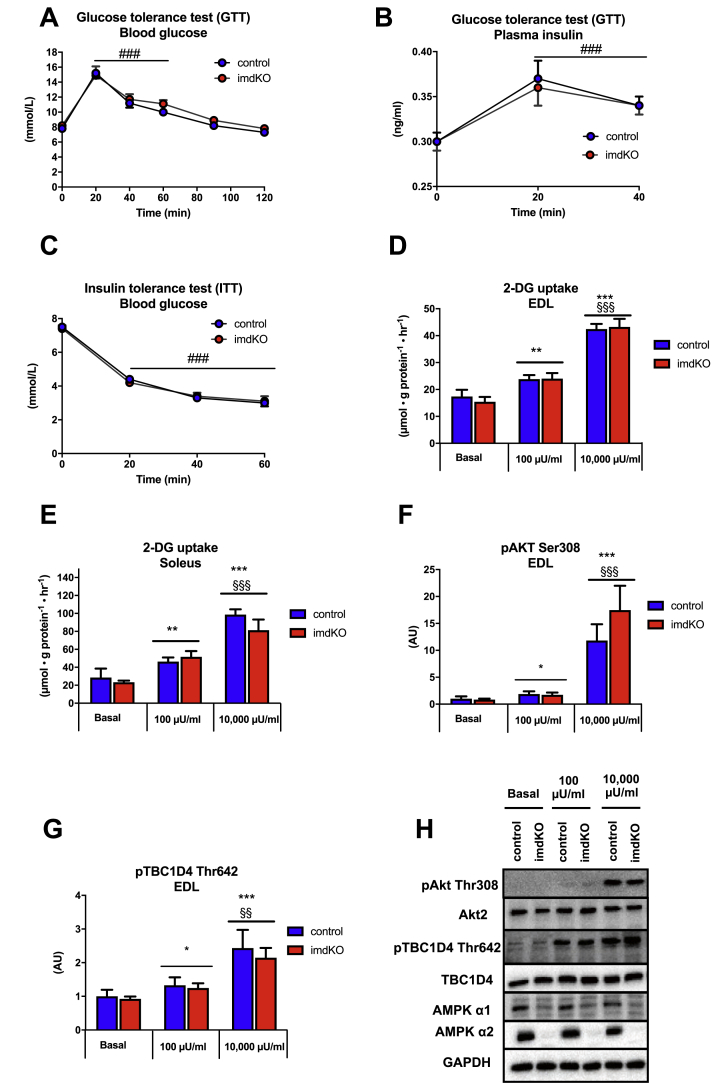
**Normal insulin action despite acute deletion of catalytic AMPK function in skeletal muscle.** A–B: 3 weeks after the last tamoxifen injection, control and AMPKα imdKO mice were fasted for 5 h before they were given an intraperitoneal injection of glucose (2 g/kg body weight) dissolved in a 0.9% saline solution. Blood was sampled from the tail vein and analyzed for glucose concentration by a glucometer before (0 min) and 20, 40, 60, 90, and 120 min after injection. Plasma insulin levels were determined at 0, 20, and 40 min by using an insulin ELISA assay (n = 10–12). C: For the insulin tolerance test (ITT), mice were fasted for 2 h, and insulin was injected intraperitoneally (1 U/kg body weight, Actrapid, Novo Nordisk, Bagsværd, Denmark). Tail vein blood glucose concentration was measured before (0 min), 20, 40, and 60 min after injection (n = 10–12). D–E: Isolated EDL and soleus muscles from control and AMPKα imdKO mice were incubated for 30 min in the absence (basal) or presence of 100 μU/ml and 10,000 μU/ml insulin, and muscle glucose uptake was determined by measuring the accumulation of intracellular [^3^H]-2-deoxyglucose (2DG) (n = 6–8). F–H: Key insulin signaling intermediates in EDL muscle from control and AMPKα imdKO mice were investigated by immunoblotting and are given as representative immunoblots. Data are given as means ± SEM. Two-way RM ANOVA was used to investigate the effect of genotype and time (GTT and ITT) or genotype and insulin concentrations (2DG uptake). ###p ≤ 0.001 for significantly different from basal (0 min). ∗∗p ≤ 0.01 and ∗∗∗p ≤ 0.001 for significantly different compared to basal. §§p ≤ 0.01 and §§§p ≤ 0.001 for significantly different from 100 μU/ml. Line indicates main effect.

Whole-body insulin action and muscle insulin sensitivity were investigated to determine whether inducible deletion of muscle AMPKα in adult mice was associated with the development of insulin resistance. Following an intraperitoneal GTT, blood glucose concentration increased similarly and showed a comparable dynamic response in the two genotypes (Figure 2A). Plasma insulin concentrations, determined before, 20 and 40 min after the glucose challenge, were also similar in the two genotypes (Figure 2B). An intraperitoneal insulin tolerance test (ITT) revealed comparable whole-body insulin action in the 2 genotypes (Figure 2C). Collectively, these data demonstrate that both whole-body insulin and glucose tolerance remain intact in mice with acute deletion of AMPK activity in skeletal muscle myofibers. Because skeletal muscle is responsible for the majority of glucose uptake during *in vivo* insulin stimulation [37], 2-DG uptake in isolated skeletal muscle was examined in the presence of a submaximal (100 μU/ml) and maximal (10,000 μU/ml) insulin concentration. Both submaximal and maximal insulin-stimulated glucose uptake in soleus and EDL muscles were comparable between AMPKα imdKO mice and control littermates (Figure 2D–E). In addition, insulin-stimulated signaling in EDL muscle at the level of Akt (Akt Thr308) and its downstream target TBC1D4 (TBC1D4 Thr642) were comparable between genotypes, suggesting that insulin signaling to GLUT4 translocation was regulated similarly in muscles from the 2 genotypes (Figure 2F–H). Overall, these findings demonstrate that acute deletion of AMPK catalytic activity in skeletal muscle does not affect whole-body insulin action or the ability of insulin to stimulate glucose uptake in isolated skeletal muscle.

### 3.4 Inducible deletion of AMPKα in adult mice impairs running performance and lowers muscle glycogen content

**Figure 3:**
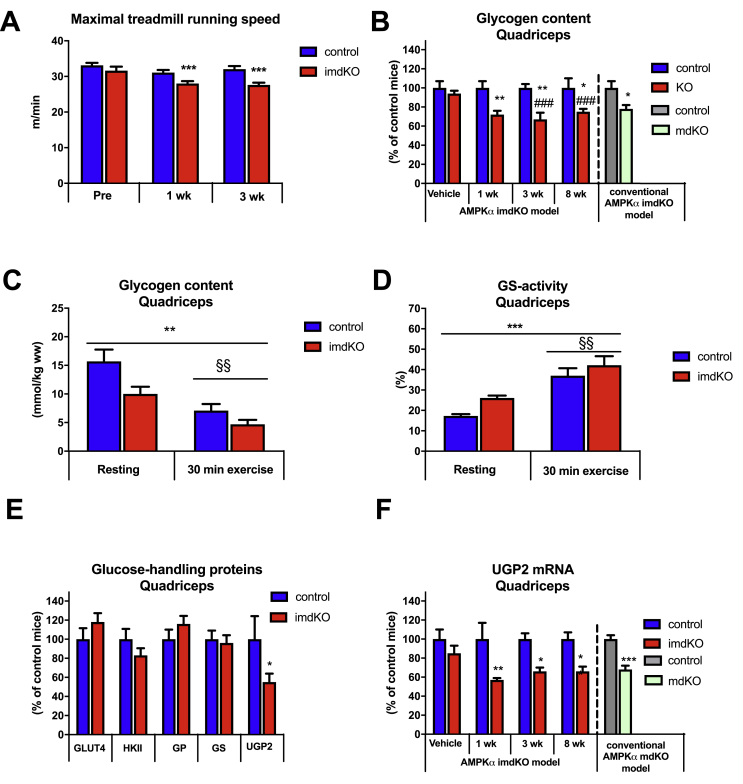
**Acute deletion of muscle AMPK impairs maximal running speed and reduces muscle glycogen content and UGP2 mRNA.** A: Maximal running speed during an incremental running test on a treadmill was assessed in control and AMPKα imdKO mice 1 and 3 weeks after last tamoxifen injection and compared to before tamoxifen treatment (pre; n = 10–20 within each group). B: Muscle glycogen content in quadriceps muscle (normalized to control mice) was measured 1, 3, and 8 weeks after tamoxifen-induced deletion of AMPKα and compared to vehicle control groups (n = 5–6 within each group). C: 3 weeks after last tamoxifen injection, muscle glycogen in quadriceps muscle from control and AMPKα imdKO mice was measured in the rested state and after 30 min of treadmill exercise at the same relative intensity (n = 8–13). D: Glycogen synthase activity was measured as fractional activity in the presence of 0.2 mM G6P and given relative to saturated conditions (8 mM G6P) (n = 8–13). E: Protein levels of GLUT4, HKII, GP, GS, and UGP2 in quadriceps muscle were measured by immunoblotting in control and AMPKα imdKO mice 3 weeks after last tamoxifen injection (n = 5–6). UGP2 mRNA content in quadriceps muscle was determined 1, 3, and 8 weeks after the last tamoxifen injection and compared to the vehicle group (sunflower oil; n = 5–6). One-way ANOVA was used for comparing 1, 3, and 8 weeks to vehicle control within AMPKα imdKO mice. An additional t-test was applied to compare AMPKα imdKO with control mice within each time point. The effect of exercise was investigated by a two-way ANOVA (C and D). Data are given as means ± SEM. ∗p ≤ 0.05, ∗∗p ≤ 0.01, and, ∗∗∗p ≤ 0.001 for effect of genotype within a time point. ###p ≤ 0.001 for difference from vehicle in AMPKα imdKO mice. §§p ≤ 0.01 for main effect of exercise. Line indicates main effect.

In accordance with observations in mouse models with embryonic and hence chronic deletion of AMPK function in skeletal muscle [9,10,16,18], a reduction in maximal treadmill running speed was also found in AMPKα imdKO mice relative to control littermates (Figure 3A). Notably, a ∼15% reduction in maximal treadmill running speed was observed as early as 1 week after tamoxifen-induced AMPKα gene deletion. This early response to AMPKα gene deletion was also found for skeletal muscle glycogen content. Thus, glycogen concentration in quadriceps muscle was decreased by ∼25% in AMPKα imdKO at 1, 3, and 8 weeks after tamoxifen treatment ended (Figure 3B).

To evaluate whether the metabolic stress induced by treadmill exercise differed between genotypes, we measured muscle glycogen concentration before and after 30 min of treadmill exercise (Figure 3C). Although resting muscle glycogen content was lower in AMPKα imdKO relative to control mice, the ability to utilize muscle glycogen (glycogen degradation) during treadmill exercise was similar between the two genotypes (Figure 3C). Moreover, the activity of the glucose-incorporating enzyme - GS - was elevated in muscle from AMPKα imdKO mice compared to control mice (Figure 3D). The content of key proteins involved in glucose uptake (GLUT4 and HKII) and glycogen degradation (glycogen phosphorylase (GP)) were similar in muscle from AMPKα imdKO and control mice. Notably, UDP-glucose pyrophosphorylase 2 (UGP2), but not GS protein content, was lower in quadriceps muscle from AMPKα imdKO mice than in control mice (Figure 3E). UGP2 is essential for glycogen synthesis because it generates UDP-glucose [38] to be incorporated into glycogen chains mainly catalyzed by GS. Analyses of the time course experiment revealed that UGP2 gene expression was reduced by ∼40% in quadriceps muscle from AMPKα imdKO mice 1 week after ended tamoxifen-induced AMPKα gene deletion (Figure 3F). This observation indicates that AMPK is involved in the regulation of UGP-2 gene expression, and we hypothesize that the concurrent lowering of muscle glycogen content in muscle from AMPKα imdKO mice is related to this change in UGP-2 expression.

These observations suggest that AMPK is required for treadmill running performance and for maintaining resting muscle glycogen content potentially through regulation of UGP2. By contrast, the ability of skeletal muscle to use glycogen as an energy source during exercise remains unaffected. Because maximal running performance differed between genotypes, the following treadmill running was performed at a relative exercise intensity (% of maximal running capacity) of each mouse.

### 3.5 AMPK is required for maintaining muscle nucleotide balance during exercise

**Figure 4:**
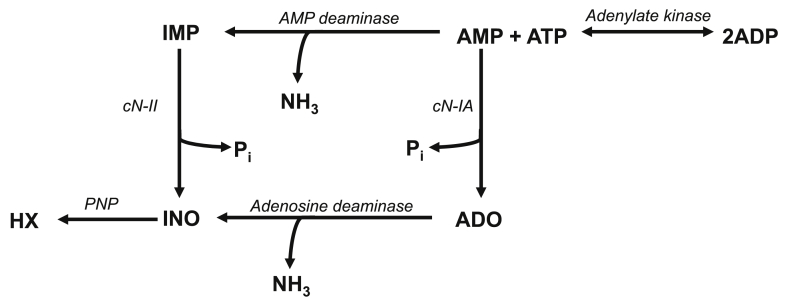
**Regulation of myocellular nucleotide pool during exercise.** Overview of cellular processes regulating cellular nucleotide balance. The increasing ATP utilization during exercise leads to ATP regeneration through the adenylate kinase reaction (2 ADP→ AMP + ATP), which increases accumulation of AMP. To avoid a large accumulation of AMP in the cell, AMP is deaminated to IMP through the enzyme AMP deaminase (AMPD). Intracellular IMP, formed during exercise, can be degraded to inosine (INO) and hypoxanthine (HX), which can leave the muscle cell, potentially causing a nucleotide loss. cN-II: Cytosolic nucleotidase II, cN-IA: Cytosolic nucleotidase IA, PNP: Purine nucleoside phosphorylase, P_i_: Inorganic phosphate, NH_3_: Ammonia.

**Figure 5:**
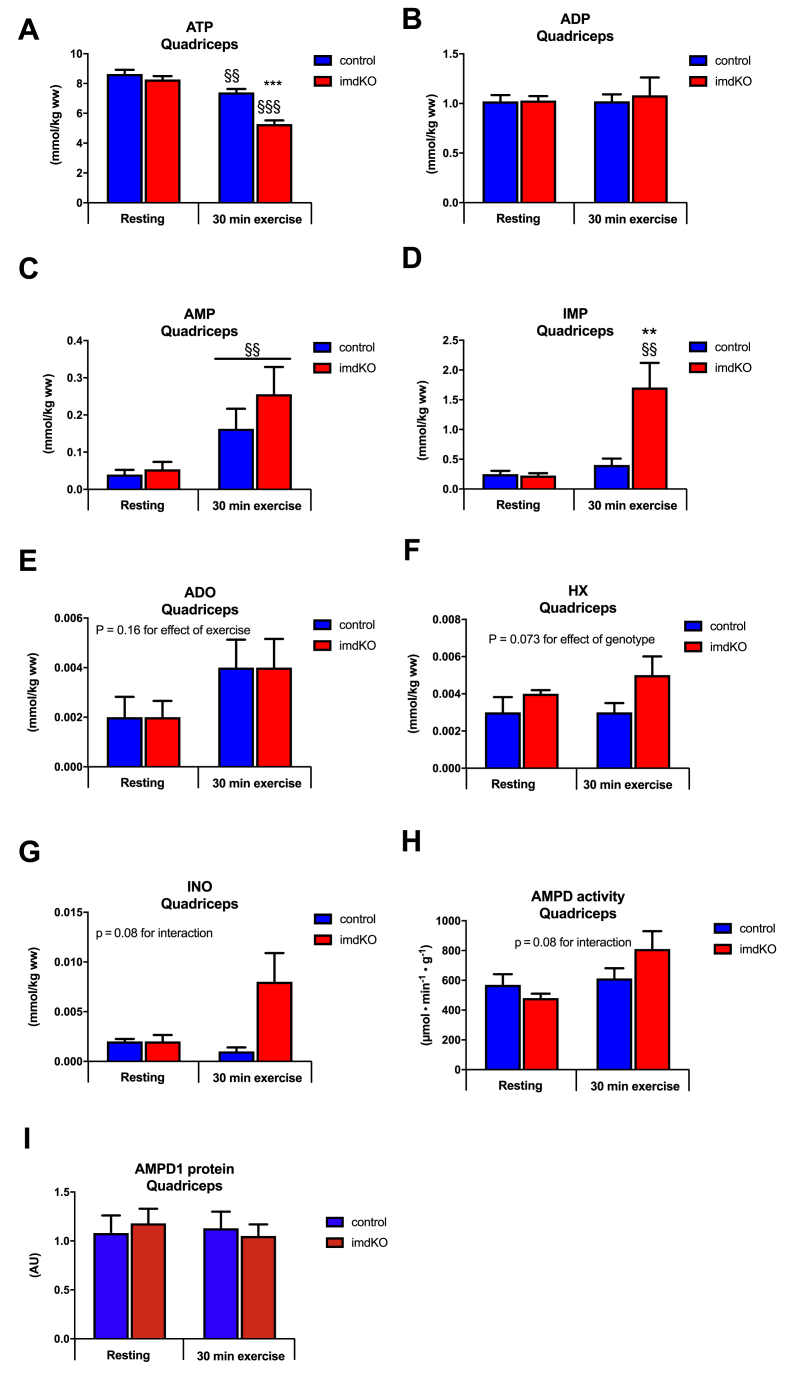
**AMPK is necessary to maintain the cellular nucleotide pool during exercise.** A–G: Control and AMPKα imdKO mice performed 30 min of treadmill exercise at the same relative intensity and were compared to corresponding resting mice. Concentration of adenosine triphosphate (ATP), adenosine diphosphate (ADP), adenosine monophosphate (AMP), inosine monophosphate (IMP), hypoxanthine (HX), adenosine (ADO), and inosine (INO) were measured in quadriceps muscle (n = 6–8). H–I: AMPD activity and AMPD1 protein content were measured in quadriceps muscle (n = 6–8). Data are given as means ± SEM. Two-way ANOVA was used for statistical analyses of genotype and exercise. ∗∗p ≤ 0.01 and ∗∗∗p ≤ 0.001 for significant effect of genotype. §§p ≤ 0.01 and §§§p ≤ 0.001 for difference from resting. Line indicates main effect.

Exercise increases the turnover of ATP in skeletal muscle, and ATP regeneration through the adenylate kinase reaction is therefore important for maintaining cellular ATP levels. To avoid the accumulation of intracellular AMP, the muscle cell deaminates AMP to IMP catalyzed by the enzyme AMPD (Figure 4). Treadmill exercise for 30 min at the same relative intensity decreased muscle ATP levels in both genotypes, but to a greater extent in AMPKα imdKO mice than in control littermates (36% vs. 14% reduction in AMPKα imdKO and control mice, respectively; Figure 5A). Although muscle ADP content remained unaffected and similar in the two genotypes (Figure 5B), a significant increase in muscle AMP content was observed in response to treadmill exercise (Figure 5C). Notably, 30 min of treadmill exercise at the same relative intensity was associated with a 7-fold increase in IMP in muscle from AMPKα imdKO mice, with no detectable increase in muscle in control littermates (Figure 5D). Lower ATP levels during exercise accompanied by accumulation of IMP suggest a disturbance in muscle energy balance when AMPKα imdKO mice perform treadmill exercise (Figure 4). Intracellular IMP, formed during exercise, can be degraded to inosine (INO) and hypoxanthine (HX), which can pass through the muscle cell membrane and hence may represent a potential loss of nucleotide precursors from the exercising muscle [39]. Adenosine (ADO) increased similarly in both genotypes in response to exercise (p = 0.16 for the main effect of exercise; Figure 5E). However, both HX (p = 0.07 for the effect of genotype) and INO (p = 0.08 for interaction between genotype and intervention) tended to be elevated in exercised muscle from AMPKα imdKO only (Figure 5F–G). Collectively, these findings suggest that AMPK is required for maintaining the skeletal muscle nucleotide balance during exercise.

The enzyme adenosine monophosphate deaminase 1 (AMPD1) is highly expressed in skeletal muscle, catalyzes the deamination of AMP to IMP, and therefore plays a key role in the purine nucleotide cycle. Notably, muscle AMPD enzyme activity (measured under saturated AMP concentrations) tended to increase in response to exercise in AMPKα imdKO and remained unchanged in muscle of control littermates (p = 0.08 for interaction between intervention and genotype; Figure 5H). However, neither a genotype-dependent difference in kinetic properties in resting muscles (Table 3 and Suppl. Fig. S3K) nor AMPD1 protein content explains this observation (Figure 5I).

**Table 3:** AMPD kinetic properties.

	WT	imdKO	p-value
V_max_ (μmol · min^−1^ · g^−1^)	479 ± 47	541 ± 83	0.35
K_m_ (mM)	0.27 ± 0,05	0.37 ± 0,13	0.46

Kinetic properties for AMPD (V_max_ and K_m_) in basal quadriceps muscle were analyzed in homogenate in the presence of 15 mM, 0.1 mM, 0.06 mM, and 0.04 mM AMP, and the formation of IMP was quantified by HPLC. Data are given as means ± SEM (n = 6–8).

Collectively these data demonstrate that AMPK is necessary for maintaining the cellular nucleotide pool during exercise. Next, we investigated whether reduced ATP regeneration in muscle from AMPKα imdKO mice was related to impaired substrate uptake and/or utilization during exercise and muscle contraction.

### 3.6 Similar substrate utilization during exercise and muscle contraction in control and AMPKα imdKO mice

**Figure 6:**
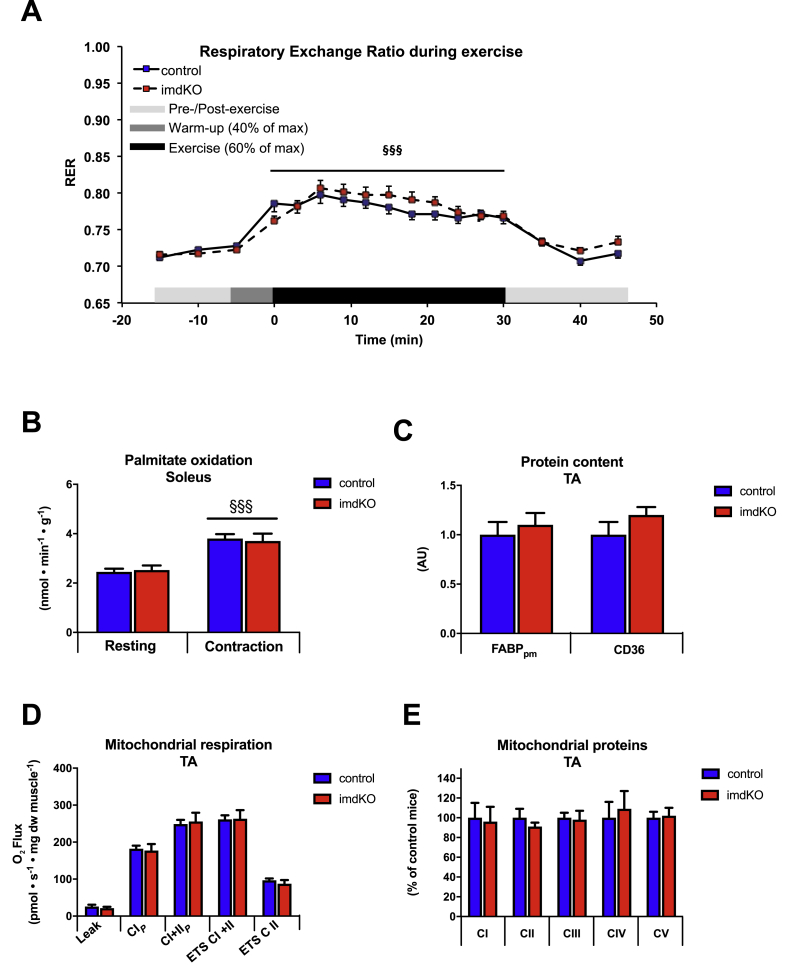
**AMPK is dispensable for regulation of muscle substrate utilization and mitochondrial function.** A: RER before, during, and after 30 min of a single treadmill exercise at approximately 60% of individual maximal running speed (n = 18–20). B: Palmitate oxidation was measured *ex vivo* in resting or contracting soleus muscles from control and AMPKα imdKO mice (n = 15–18). C. Protein levels of plasma membrane fatty acid binding protein (FABPpm) and cluster of differentiation (CD) 36 were analyzed in TA muscle by immunoblotting (n = 8–13). D: Mitochondrial respiration rates were measured during cumulative addition of substrates in permeabilized TA fibers (n = 9–12). Abbreviations: CI_P_: Maximal complex I respiration, CI+II_P_: Maximal complex I+II linked respiration (capacity for oxidative phosphorylation), ETS (CI+II): Electron transport system capacity (uncoupled respiration) through complex I and II, ETS (CII): Electron transport system capacity through complex II. E: Protein levels of mitochondrial subunits for complex I, II, III, IV, and V in TA muscle were determined by immunoblotting (n = 17–18). Data are given as means ± SEM. The effect of exercise and genotype was investigated by two-way ANOVA. §§§p ≤ 0.001 for difference from resting. Line indicates main effect.

Substrate utilization in control and AMPKα imdKO mice during treadmill exercise was investigated at the same relative intensity (60% of individual maximal running speed). RER before exercise was comparable in the 2 genotypes (∼0.72) and increased similarly during 30 min of treadmill exercise (averaged 0.78 ± 0.01 and 0.79 ± 0.01 for control and AMPKα imdKO mice, respectively; Figure 6A). Upon cessation of exercise, RER declined to pre-exercise levels with no difference between genotypes. These findings suggest that exercise at the same relative intensity exerts a similar metabolic response in control and AMPKα imdKO mice. In accordance, the measurement of exogenous palmitate oxidation in isolated soleus muscles revealed that FA oxidation was similar between genotypes at rest and increased to the same extent in response to electrically stimulated muscle contractions (Figure 6B). Impaired FA oxidation in mice with chronic deletion of AMPK activity in skeletal muscle (AMPKα mdKO) has been associated with reduced expression of FA handling transport proteins (CD36 and FABPpm) [16,40]. However, the protein content of these FA transporters was similar in control and AMPKα imdKO mice (Figure 6C and Suppl. Fig. S3H).

The literature on AMPK-deficient mouse models has reported impaired mitochondrial function and/or expression of mitochondrial proteins [9,15,41]. To investigate the effect of AMPKα deletion in adult mice on mitochondrial function, we measured mitochondrial respiration rates in permeabilized fibers from TA muscle. Mitochondrial respiration was similar in the two genotypes when analyzed in the successive presence of malate + glutamate + pyruvate (Leak), ADP (CI_*P*_), succinate (CI+II_*P*_), FCCP (ETS CI+II), and rotenone (ETS CII) (Figure 6D). These observations are in line with similar protein levels of subunits in complex I–V of the mitochondrial electron transport chain (Figure 6E and Suppl. Fig. S3I).

Collectively, these findings suggest that the phenotypic trait in the AMPK-deficient mouse models in the literature is a consequence of chronic deletion that probably does not reflect the consequences of lacking AMPK activity acutely in skeletal muscle.

### 3.7 Normal muscle glucose uptake during *in vivo* exercise and *ex vivo* muscle contractions in AMPKα imdKO mice

**Figure 7:**
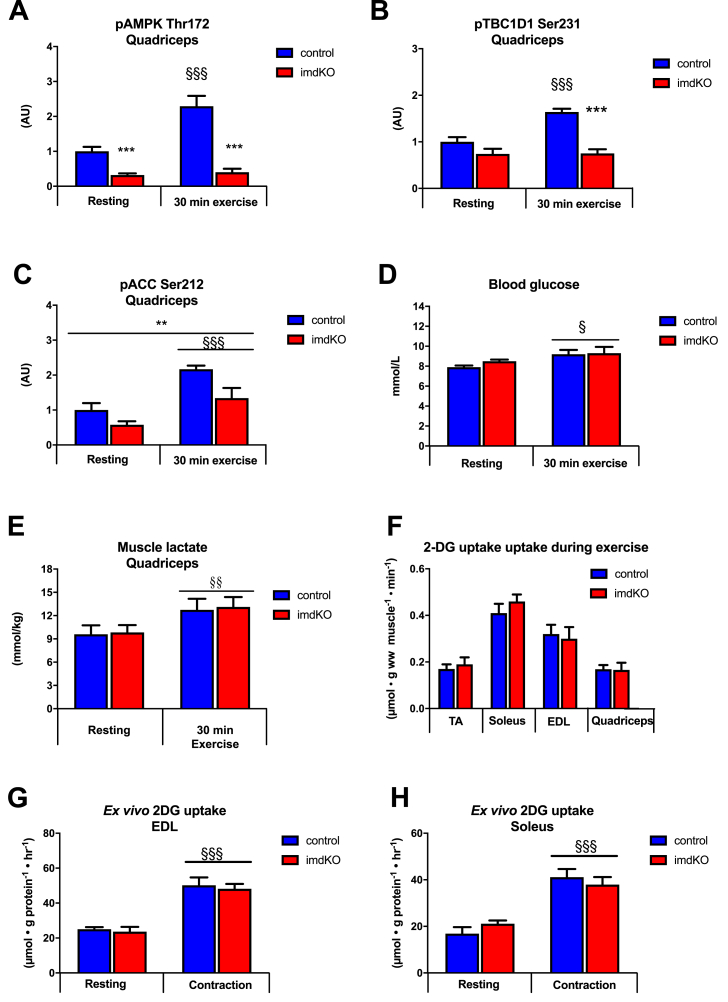
**AMPK is not required for exercise and contraction-stimulated glucose uptake in skeletal muscle.** A–C: Control and AMPKα imdKO mice were either rested or performed 30 min treadmill exercise at the same relative intensity. Phosphorylation of AMPKα Thr172, TBC1D1 Ser231, and ACC Ser212 was determined in quadriceps muscle by immunoblotting (n = 8–13). D–E: Blood glucose concentration and muscle lactate concentration in quadriceps muscle measured under resting conditions and after 30 min of treadmill exercise (n = 8–13). F: Muscle glucose uptake during 30 min of treadmill exercise was measured in TA, soleus, EDL, and quadriceps muscle from control and AMPKα imdKO mice (n = 8–13). G–H: Isolated EDL and soleus muscle from control and AMPKα imdKO mice were electrically forced to contract, and glucose uptake was measured in resting and contracting muscles (n = 4–8). Data are given as means ± SEM. The effect of genotype and exercise/muscle contraction was investigated by two-way ANOVA. ∗∗p ≤ 0.01 and ∗∗∗p ≤ 0.001 for significant effect of genotype. §p ≤ 0.05, §§p ≤ 0.01 and §§§p ≤ 0.001 for difference from resting. Line indicates main effects.

Treadmill exercise for 30 min increased AMPK phosphorylation at Thr172, a surrogate marker for AMPK activation, in quadriceps muscle from control mice; this effect was absent in AMPKα imdKO mice (Figure 7A and Suppl. Fig. S3J). Similarly, treadmill exercise increased phosphorylation of TBC1D1 Ser231 in quadriceps muscle from control but not in muscle from AMPKα imdKO mice (Figure 7B). This observation was not due to a difference in TBC1D1 protein levels because these were similar in quadriceps muscle from control and AMPKα imdKO mice. In the present study, we observed generally lowered phosphorylation of ACC Ser212 in muscle from AMPKα imdKO mice, and the ability to increase ACC Ser212 phosphorylation in response to exercise was maintained (Figure 7C and Suppl. Fig. S3J). This finding may either suggest that other kinases than AMPK are capable of phosphorylating ACC Ser212 during exercise or that AMPK-induced phosphorylation of ACC Ser212 occurs in the non-muscle cells in the crude muscle sample preparations. Studies investigating contraction-stimulated and exercise-stimulated glucose uptake in skeletal muscle by using other AMPK-deficient mouse models have suggested no or a partial role of AMPK [9,12,[14], [15], [16],^42^]. In conventional AMPKα mdKO mice, exercise is associated with markedly elevated blood glucose levels (increase from ∼7 mM to ∼12.0 mM during exercise) [16]. However, in this study, we found that 30 min of treadmill exercise resulted in a minor increase in blood glucose levels in both control and AMPKα imdKO mice that occurred concomitantly with a similar increase in muscle lactate content (Figure 7D–E).

Glucose uptake in TA, soleus, EDL, and quadriceps muscle during exercise was similar between control and AMPKα imdKO mice (Figure 7F). These data demonstrate that muscle glucose uptake during *in vivo* exercise is not compromised by muscle-specific deletion of AMPK catalytic activity in adult mice. To further illuminate this matter, contraction-stimulated 2-DG uptake was assessed in isolated EDL and soleus muscles from control and AMPKα imdKO mice. During tetanic muscle contractions, glucose uptake increased to a similar extent in isolated soleus and EDL muscles from control and AMPKα imdKO mice (Figure 7G–H). Muscle force development during this electrical stimulation protocol showed no difference between genotypes in EDL muscle (Suppl. Fig. S5A) but was significantly lower (∼23% lower at all time points) in soleus muscle (p = 0.05) from AMPKα imdKO mice than from control littermates (Suppl. Fig. S5B). Thus, although force development was generally lower for soleus muscle from AMPKα imdKO mice, the decline in force development over time (fatigue) showed a similar pattern in both genotypes. Force development during electrically stimulated single twitch stimulation resulted in comparable force development (Suppl. Fig S5C). *Ex vivo* contractions increased phosphorylation of AMPKα Thr172 in control EDL and soleus muscles, and phosphorylation of AMPKα Thr172 in AMPKα imdKO muscle at rest was reduced and increased modestly in soleus muscle in response to contractions (Suppl. Figs 5D–E and S5J). Both basal and contraction-stimulated phosphorylation of AMPK downstream targets, ACC Ser212 and TBC1D1 Ser231, were reduced in EDL muscle from AMPKα imdKO mice (Suppl. Figs 5F–G and S5J). However, in response to contractions, phosphorylation of ACC Ser212 and TBC1D1 Ser231 in soleus muscle increased similarly in both genotypes (Suppl. Figs S5H–J). ACC and TBC1D1 protein levels in soleus were comparable in control and AMPKα imdKO mice (data not shown), and TBC1D1 protein content was lower (∼25%) in EDL muscle from AMPKα imdKO mice than from control mice (data not shown).

Collectively, these data demonstrate that AMPK is dispensable for contraction-stimulated glucose uptake in skeletal muscle and that the signaling axis leading to increased glucose uptake, at least in glycolytic quadriceps and EDL muscles, can be mediated independently of TBC1D1 phosphoregulation.

## 4 Discussion

The central role of AMPK as a gatekeeper in the regulation of skeletal muscle metabolism is mainly based on AMPK-deficient mouse models with chronic lack of AMPK function. However, results derived from such models are potentially biased by confounding adaptations due to the lifelong AMPK deficiency (including embryonic development), as emphasized by marked defects in metabolic proteins, mitochondrial function, and extreme exercise intolerance in some of these conventional models. In an attempt to minimize the influence of these confounding factors and study the direct and acute effects of AMPK deficiency, we generated a new transgenic mouse model with tamoxifen-inducible muscle-specific deletion of catalytic AMPK activity in adult mice (AMPKα imdKO).

The literature has reported that whole-body deletion of AMPKα2 leads to greater degradation of ATP in skeletal muscle during exercise and is associated with accumulation of AMP and IMP [43]. However, in that study, the whole body AMPKα2 KO mice performed exercise at the same absolute intensity as the control littermates. Today, we know that these findings were biased because the maximal running capacity of this KO model is reduced compared to WT control mice. Therefore, these observations may be related to a difference in exercise workload between genotypes rather than a direct consequence of a lack in AMPK activity. Notably, Lee-Young and co-workers reported accelerated ATP degradation but similar AMP concentrations during exercise in muscle from AMPKα2 KD mice compared to control mice, although exercise was performed at the same relative exercise intensity [10]. The study also reported a marked impairment in mitochondrial respiratory capacity (∼32 and 50% impairment in complex I and IV activities, respectively); therefore, we speculate that the accelerated ATP depletion in muscle from AMPKα2 KD mice was the result of a reduced capacity for aerobic ATP repletion due to mitochondrial dysfunction. In the present study, ATP degradation was also accelerated in muscle from AMPKα imdKO mice, despite intact mitochondrial function and that these mice were running at the same relative exercise intensity. This suggests that AMPK activation during exercise is necessary for maintaining myocellular ATP levels during exercise. The greater ATP degradation during exercise in combination with lower muscle glycogen content in AMPKα imdKO mice may also explain the lower maximal running speed observed in these mice. Compared with the literature, these observations were independent of muscle glucose uptake, substrate utilization, and mitochondrial function.

The role of AMPK in the regulation of substrate utilization during exercise has been investigated in different mouse models with chronic deletion of AMPK activity [9,16,17,44]. AMPKβ1β2M−KO mice show increased reliance on FA oxidation during exercise (decreased RER) [9], but direct interpretation of this observation is compromised by extreme exercise intolerance (∼57% reduction in maximal running speed) and hence a dramatically lower absolute running speed during treadmill exercise. By contrast, deletion of both AMPKα isoforms (AMPKα mdKO) or the upstream kinase LKB1 (LKB1 KO) in skeletal muscle results in increased reliance on glucose utilization during exercise (increased RER) [16,17]. This may be a consequence of impaired FA oxidation due to lowered expression of fat transport proteins (e.g., CD36, FABPpm) and lowered mitochondrial capacity/enzyme activity reported for these mice [[15], [16], [17]]. In this study, we observed normal substrate utilization during *in vivo* exercise and *ex vivo* contractions of isolated muscles, and intact expression of FA transporter proteins and mitochondrial respiratory function. Collectively, these observations support the notion that alterations in substrate utilization in mouse models with chronic deletion of AMPK activity are due to persistent alterations in the protein expression profile, mitochondrial function, or extreme exercise intolerance rather than the consequences of lacking acute AMPK-related regulation.

One of the proposed roles of AMPD is to prevent a large increase in ADP by removing AMP, hereby favoring ATP formation by the adenylate kinase reaction [45]. Thus, we interpret the trend toward increased AMPD activity and massive formation of IMP in skeletal muscle from AMPKα imdKO mice during exercise as a protective mechanism to avoid accumulation of AMP. *In vitro* studies have reported that AMPD activity is enhanced during muscle contractions in correspondence with elevated H^+^ as a result of lactate formation [46,47]. However, muscle lactate concentration increased similarly in response to exercise in control and AMPKα imdKO mice, suggesting that factors rather than muscle H^+^ accumulation contribute to the elevated AMPD activity in muscle from AMPKα imdKO mice. For *in vitro* AMPD activity measurements, the muscle homogenate was diluted ∼500 fold for the assay procedure, probably eliminating the possible influence of the soluble factors in the muscle. This suggests the presence of a regulation *in vivo* during exercise in AMPKα imdKO mice that is preserved during *in vitro* AMPD activity measurements. Phosphatase treatment has been demonstrated to alter the affinity of AMPD for its substrate AMP while the V_max_ remains unchanged [30]. This finding suggests that allosteric regulations other than protein phosphorylation have increased maximal AMPD activity in muscle from exercising AMPKα imdKO mice in the present study.

Although AMPK activation by pharmacological means demonstrates that AMPK is sufficient to increase muscle glucose uptake [[6], [7], [8]], the proposed necessary role of AMPK in the regulation of contraction-stimulated muscle glucose uptake has been studied intensively in various transgenic mouse models with conflicting findings. Some studies have reported intact glucose uptake during *ex vivo* muscle contractions [13,15,19,20,22]; other studies have reported a partially decreased ability to increase muscle glucose uptake in response to contractile activity under some experimental conditions [9,[11], [12], [13], [14], [15]]. Moreover, glucose uptake during exercise is impaired in AMPKβ1β2M−KO mice but remains intact in AMPKα mdKO and LKB1-KO mice [9,16,17]. In this study, we used an inducible AMPKα KO mouse model and observed that AMPK is dispensable for regulating glucose uptake in response to *in vivo* exercise and *ex vivo* muscle contractions.

TBC1D1 has been suggested to be involved in AMPK-mediated signaling that regulates GLUT4 translocation to increase muscle glucose uptake during muscle contractions [48]. Other studies have reported reduced phosphorylation of TBC1D1 at Ser231 in muscle of AMPK-deficient mice in response to muscle contractions [9,16,[49], [50], [51]]. Moreover, muscle from TBC1D1-deficient mouse models shows reduced contraction-stimulated and exercise-stimulated glucose uptake [[52], [53], [54]], suggesting that the AMPK-TBC1D1 signaling axis is required for regulating glucose uptake during exercise. However, the TBC1D1 KO mouse model is associated with impaired GLUT4 expression in skeletal muscle, complicating the overall interpretation of those findings. Overexpression of TBC1D1 mutated to alanine at 4 phosphorylation sites in skeletal muscle (Ser231Ala, Thr499Ala, Thr590Ala, and Ser621Ala) seems not to affect GLUT4 protein content and remains associated with a marked reduction (22%) in contraction-stimulated glucose transport [55]. By contrast, mutation of a single phosphorylation site on TBC1D1 (Ser231Ala) does not compromise contraction-stimulated and exercise-stimulated glucose uptake [56]. In the present study, contraction-stimulated TBC1D1 phosphorylation at Ser231 did not increase in glycolytic muscles (quadriceps and EDL) from AMPKα imdKO mice, albeit muscle glucose uptake increased similarly in both genotypes. Recently, our research group clarified these seemingly discrepant findings of AMPK-TBC1D1 signaling in regard to contraction-stimulated muscle glucose uptake. Thus, we convincingly show that AMPK and TBC1D1 are necessary and important for maintaining elevated glucose uptake in skeletal muscle in the immediate period after, but not during, exercise and contraction [57]. Altogether, these findings support the concept that glucose uptake can be regulated independently of TBC1D1 phosphorylation at Ser231, and we speculate that muscle contractile activity leads to an activation of a broad range of intracellular signaling events that promote glucose uptake and fat oxidation during exercise, independently of AMPK.

All experiments in the present study were performed at room temperature and hence below mouse thermoneutrality. Recent evidence suggests that mild cold stress induced by ambient housing may confound the experimental outcome in mice [58]. However, McKie and colleagues observed that exercise-induced gene expression and AMPK activation in muscle were comparable between experiments performed at room temperature and thermoneutrality [59]. This indicates that at least the acute exercise responses in skeletal muscle in this study would also have been observed at thermoneutrality.

Notably, the promptly reduced muscle glycogen content (1 week after tamoxifen treatment ended) in AMPKα imdKO mice was accompanied by a corresponding reduction in UGP2 gene expression. Support for a direct link between AMPK activation and UGP2 mRNA has been provided by the transcriptomic profiling (microarray analysis) of muscles from AMPKγ3^R225Q^ transgenic mice, AMPKγ3^−/−^ knockout mice, and AICAR-treated wild-type mice [60]. Thus, increased AMPK activity, either by introducing an activating AMPKγ3 mutation (AMPKγ3^R225Q^) or AMPK activation by AICAR, induced an increase in UGP2 mRNA, and genetic deletion of AMPKγ3 (AMPKγ3^−/−^) induced a corresponding reduction. Furthermore, the AMPKγ3 gain of function mutation in pig muscle (AMPK*γ*3^R200Q^) leads to a 3-fold increase in UGP2 protein levels [61]. Based on mutational cell experiments, GS is considered rate-limiting for glycogen synthesis, and UGP2 is generally considered to play a minor role in glycogen storage [62]. However, we speculate that UGP2 may contribute to glycogen storage capacity by promoting glucose flux toward GS and may explain the lower muscle glycogen content observed in AMPKα imdKO mice.

In conclusion, we have generated a new mouse model with inducible skeletal muscle-specific deletion of the catalytic AMPKα subunits in adult mice that allows the study of the direct effect(s) of AMPK in muscle metabolism. Acute deletion of AMPK activity in adult mouse muscle reveals that intracellular mediators other than AMPK are sufficient to regulate glucose uptake and substrate utilization in response to exercise and muscle contractions. However, AMPK is central for maintaining cellular nucleotide balance during exercise, because increased deamination of AMP to IMP is observed in muscle from AMPKα imdKO mice during exercise. Moreover, acute deletion of muscle AMPKα in adult mice promptly reduces muscle glycogen content and lowers UGP2 expression. These observations may explain the lower maximal treadmill running speed in AMPKα imdKO observed 1 week after AMPKα gene deletion.

## Funding sources

The study was supported by grants (FSS 8020–00288/6110 00498B) from the 10.13039/100008392Danish Council for Independent Research, Medical Sciences (to J.F.P.W.), the 10.13039/501100009708Novo Nordisk Foundation (NNF16OC0023046) (to J.F.P.W.), and the 10.13039/501100003554Lundbeck Foundation (R266-2017-4358) (to J.F.P.W.). This work was supported by a research grant (to R.K. and A.M.F.) from the 10.13039/100015223Danish Diabetes Academy, which is funded by the 10.13039/501100009708Novo Nordisk Foundation, grant number NNF17SA0031406.

## Appendix A Supplementary data

Supplementary Figures 3, 4 and 5 Supplementary Figures 3, 4 and 5

Supplmental 1: Primer and probe sequences used for real time PCR Supplmental 1: Primer and probe sequences used for real time PCR

Supplemental 2: List of antibodies Supplemental 2: List of antibodies
