# Supplementary material for: Inducible deletion of skeletal muscle AMPKα reveals that AMPK is required for nucleotide balance but dispensable for muscle glucose uptake and fat oxidation during exercise
Source: Mol Metab. 2020 Jun 3;40:101028. doi: 10.1016/j.molmet.2020.101028 (PMC7356270; doi:10.1016/j.molmet.2020.101028)

Supplemental 3

S3A

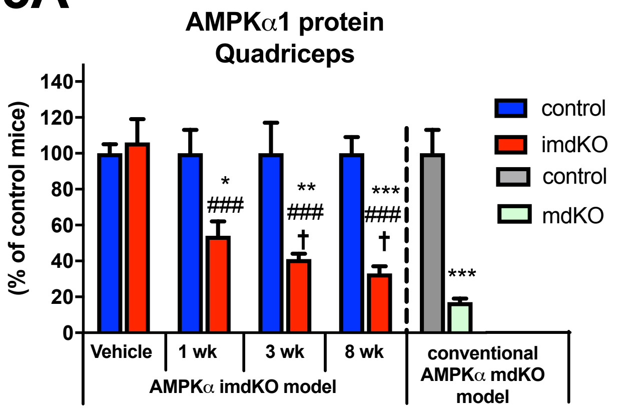

S3B

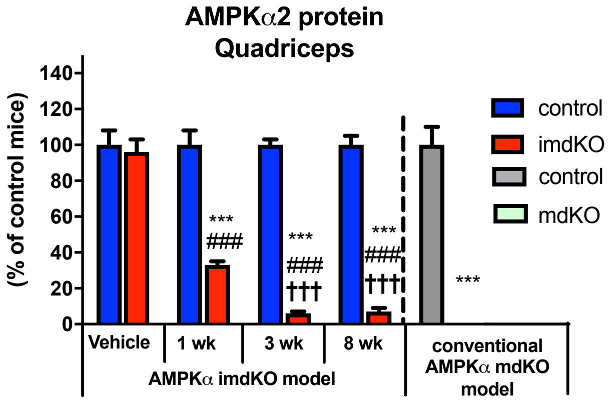

S3C

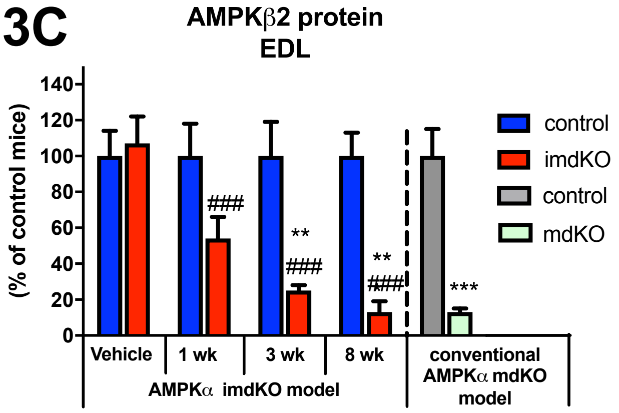

S3D

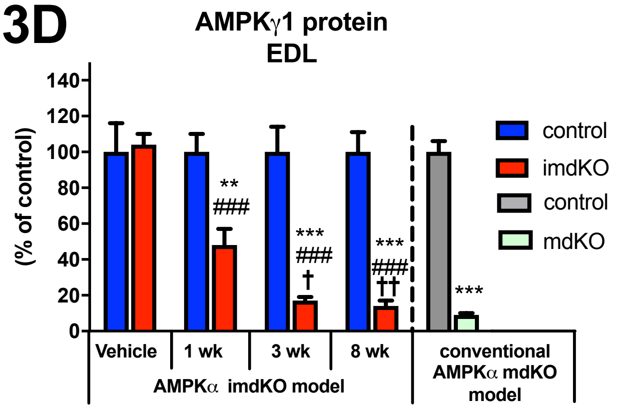

S3E

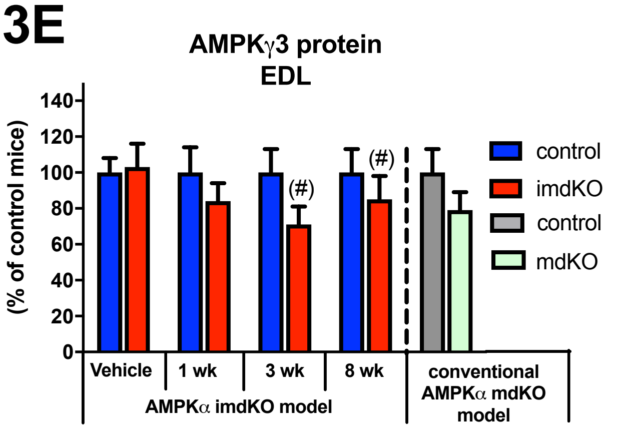

Supplemental 3

S3F

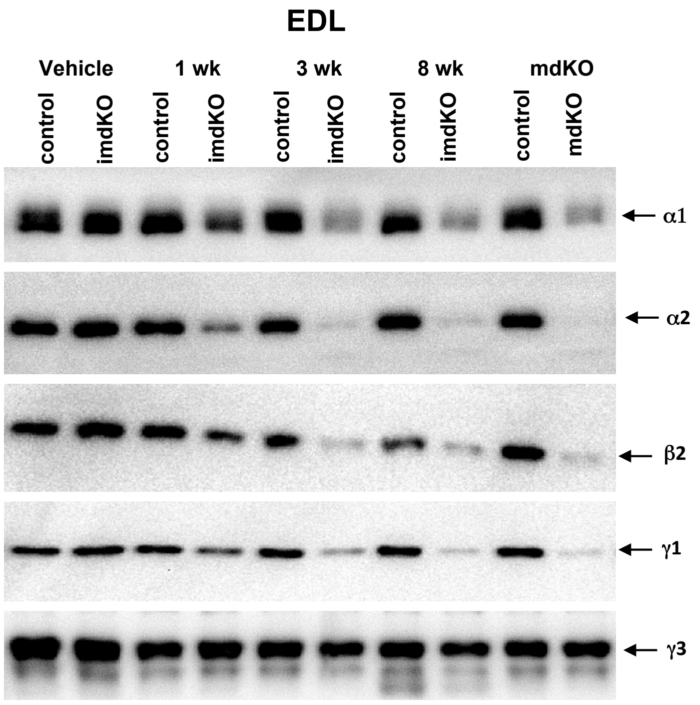

S3G

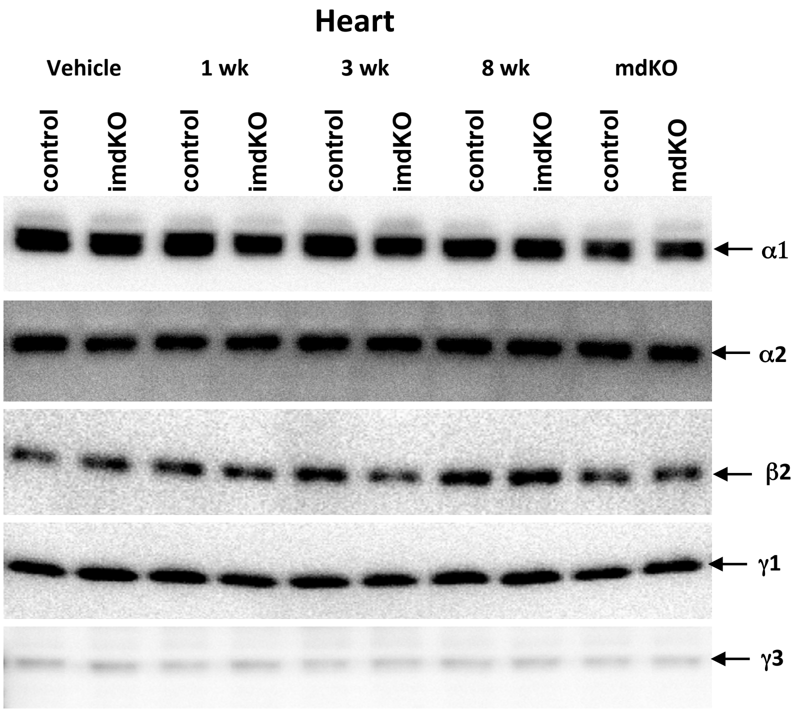

S3H

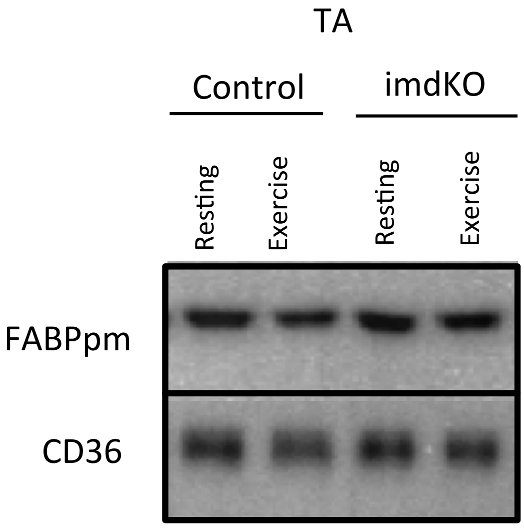

S3I

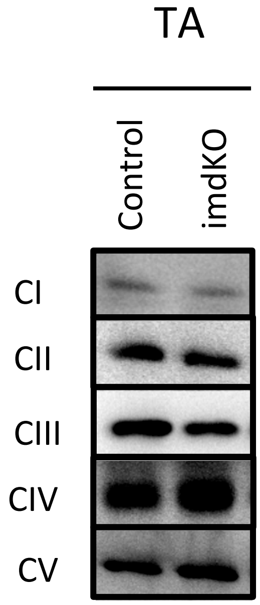

S3J

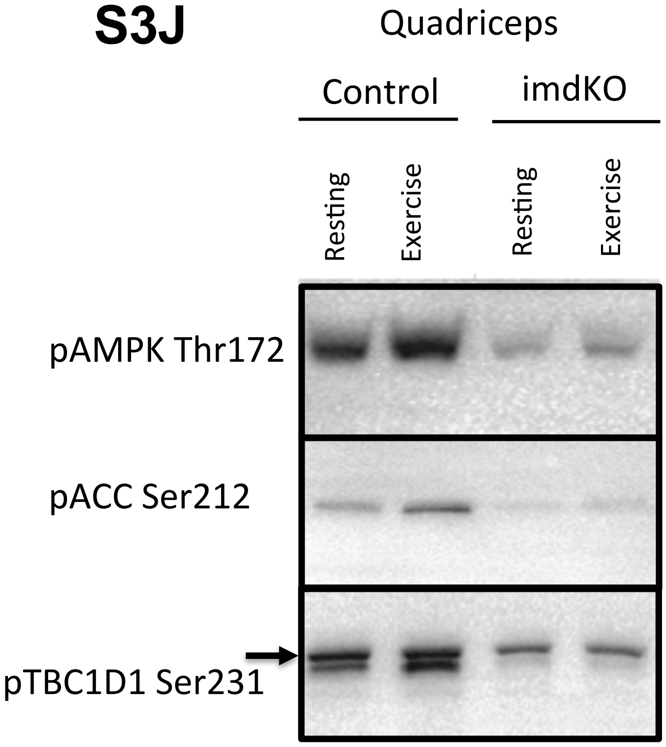

S3K

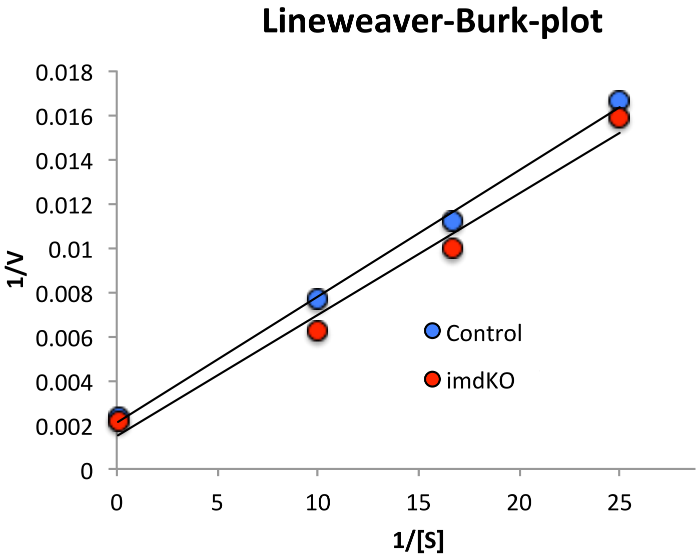

Supplemental 4: Resting metabolism

4A

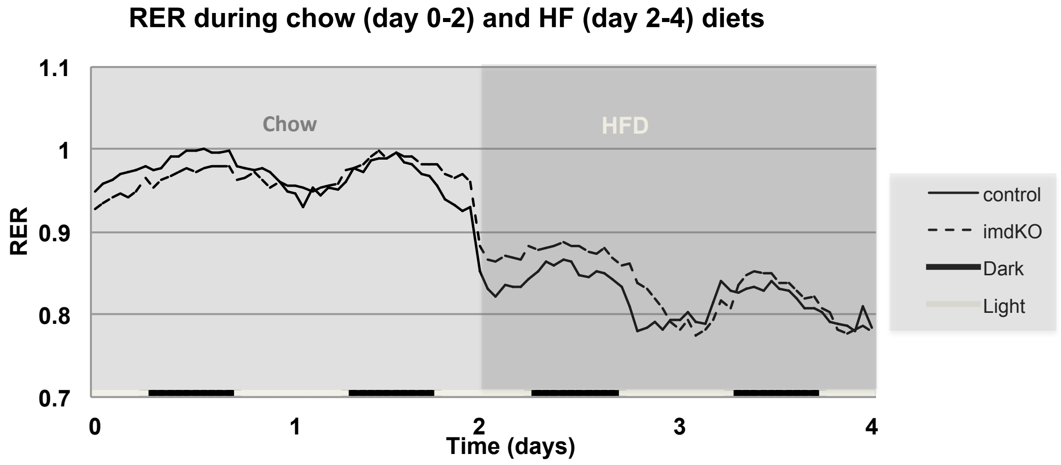

4B

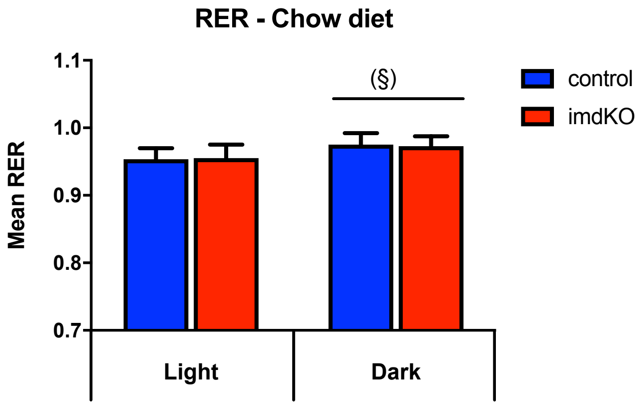

4C

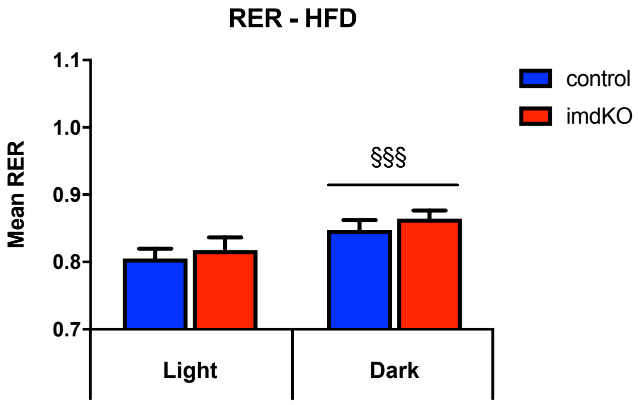

Supplemental 4: Resting metabolism

4D

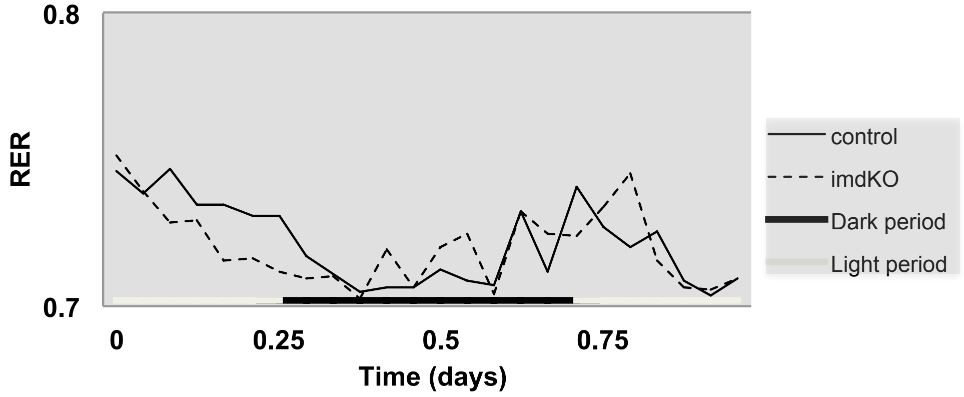

4E

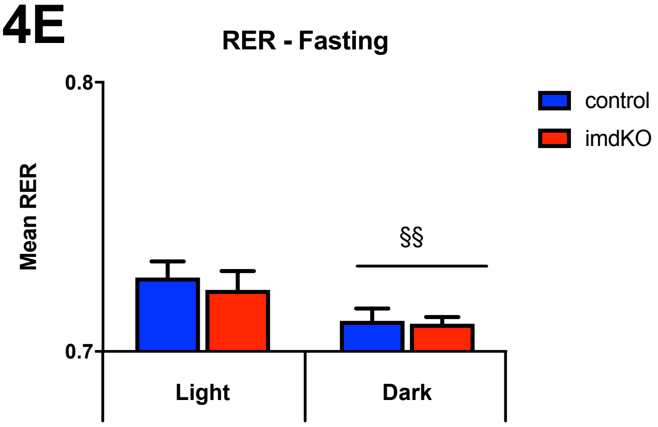

Supplemental 4: Resting metabolism

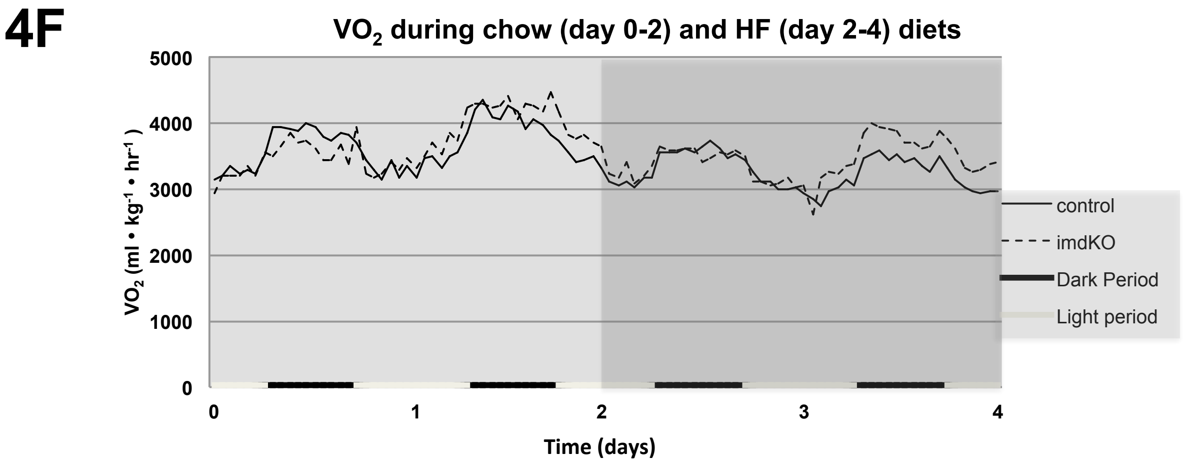

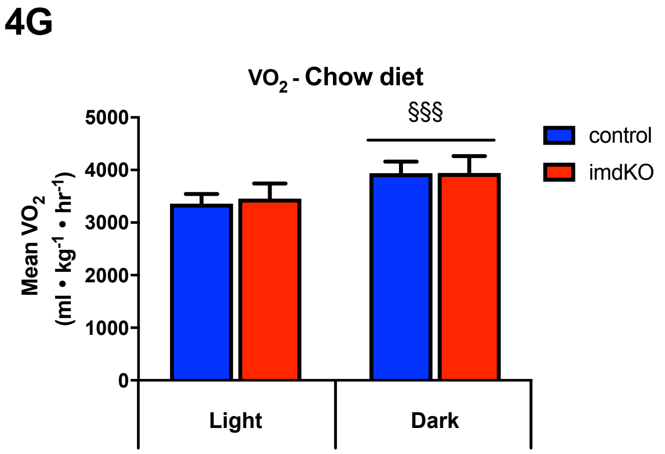

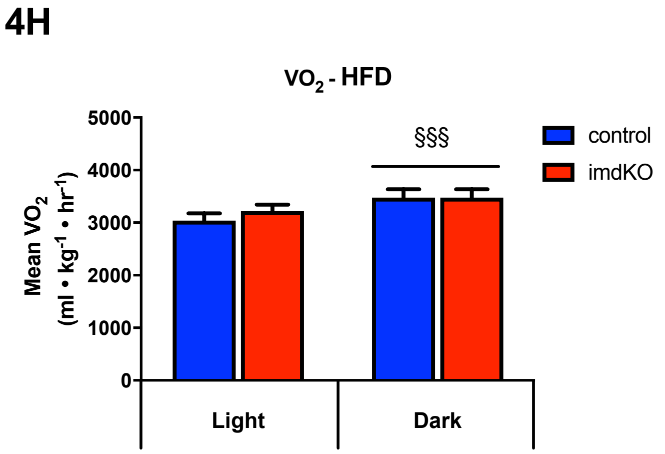

Supplemental 4: Resting metabolism

4I

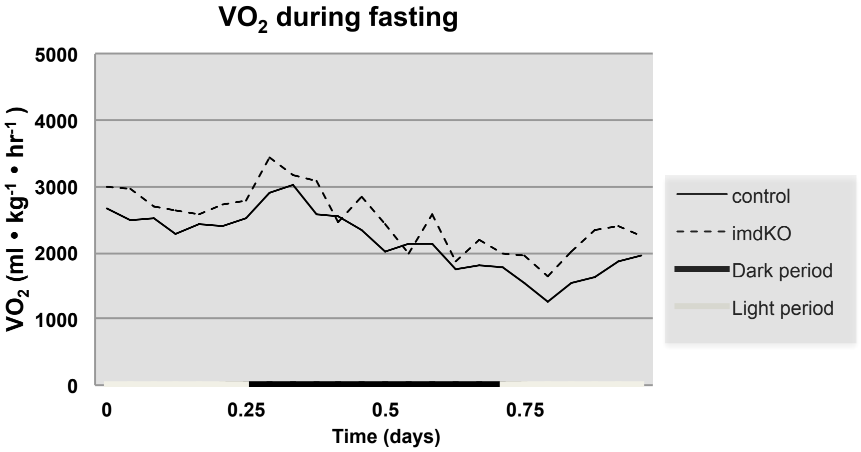

4J

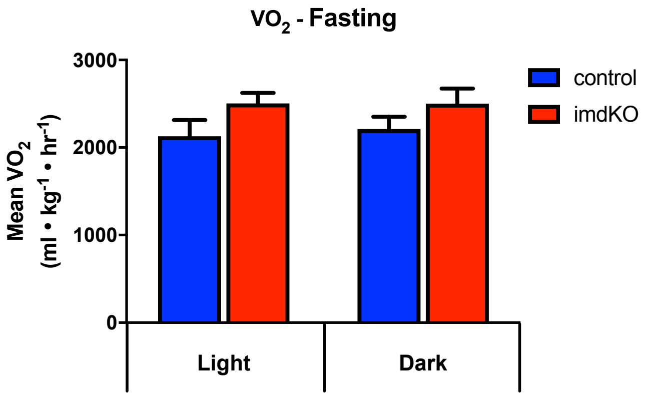

Supplemental 4: Resting metabolism

4K

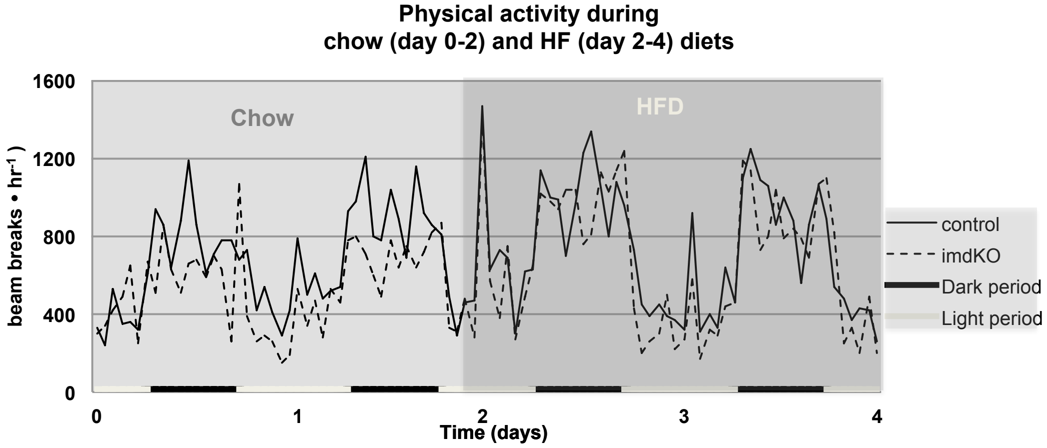

4L

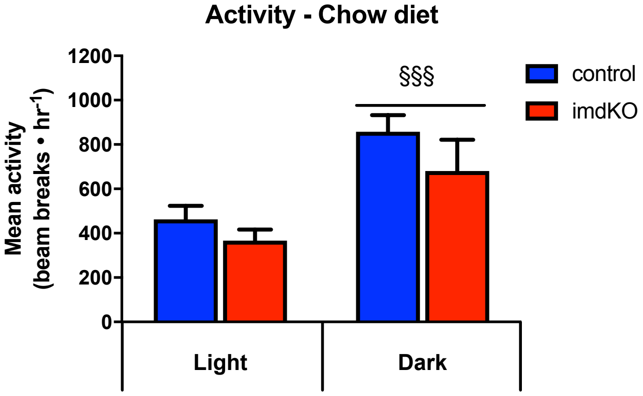

4M

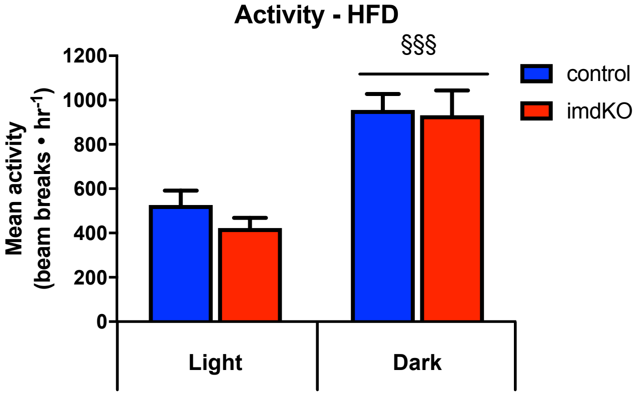

Supplemental 4: Resting metabolism

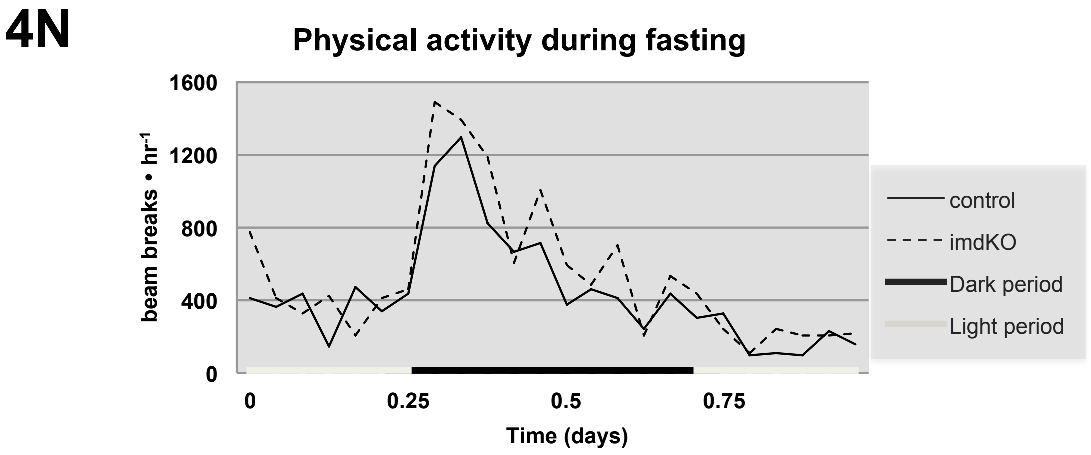

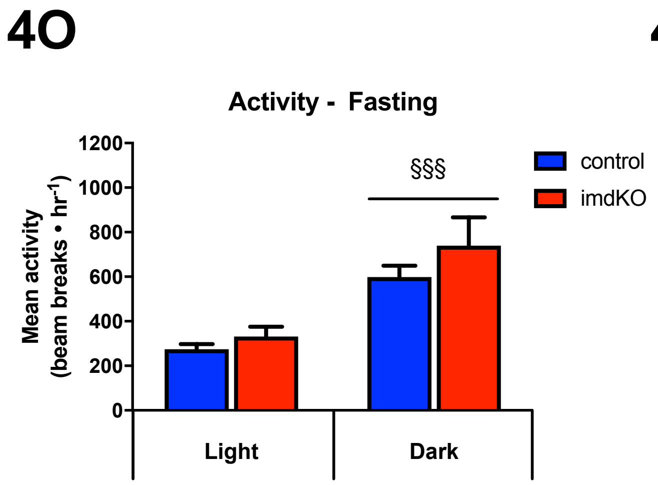

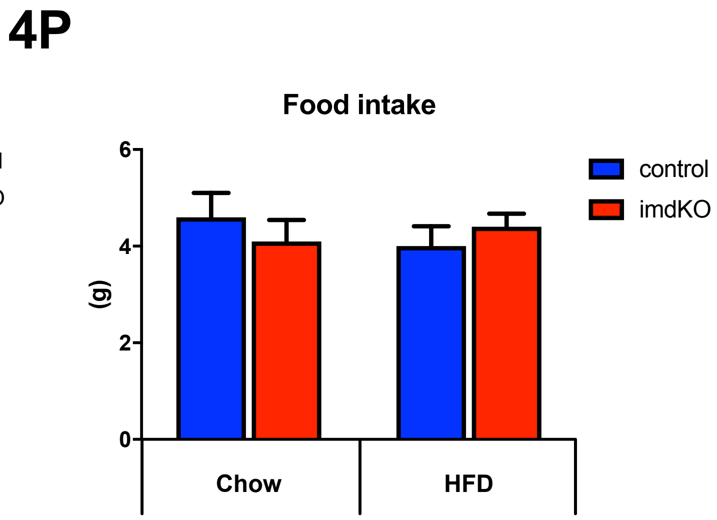

Supplemental 5

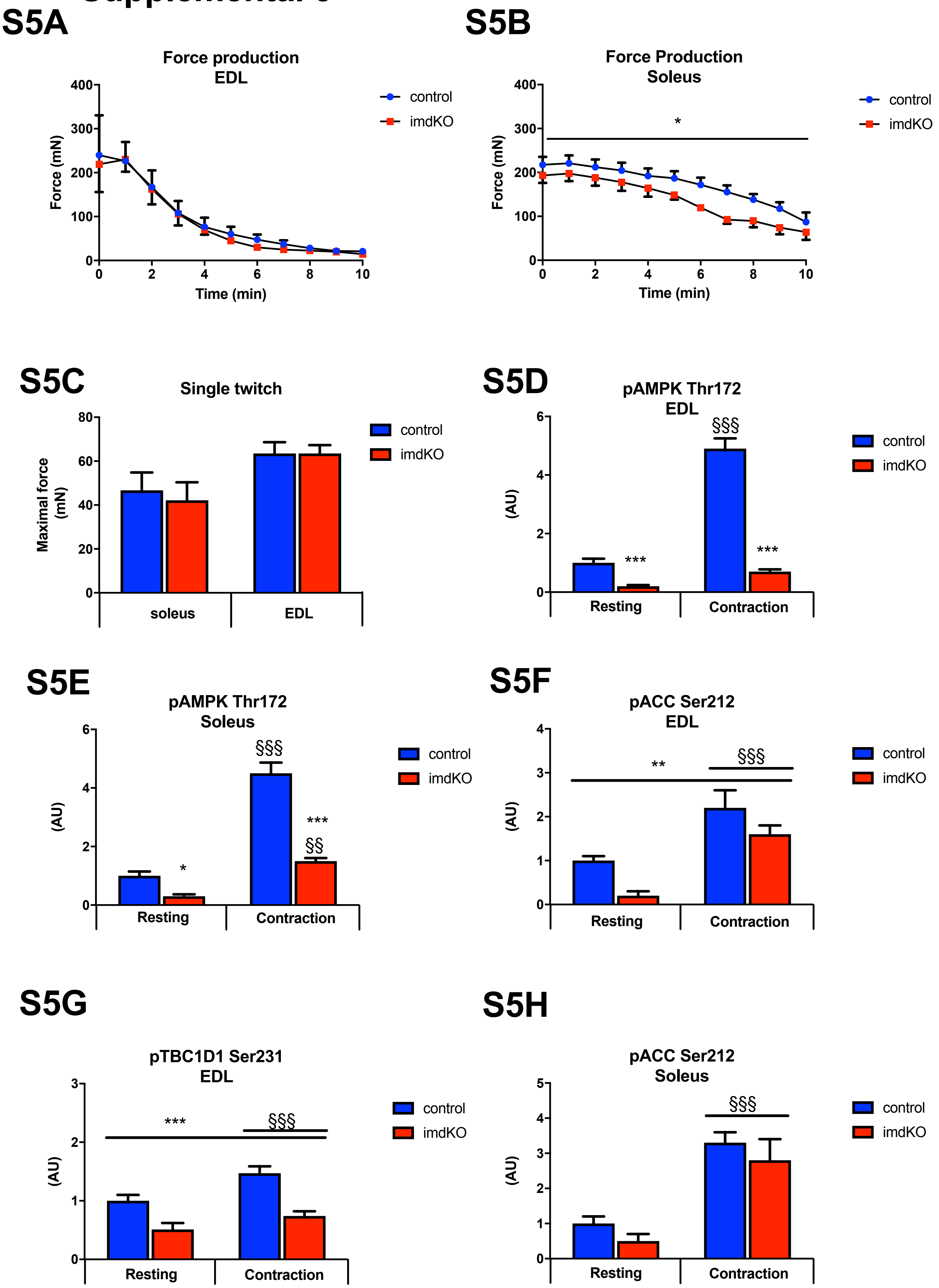

Supplemental 5

S5I

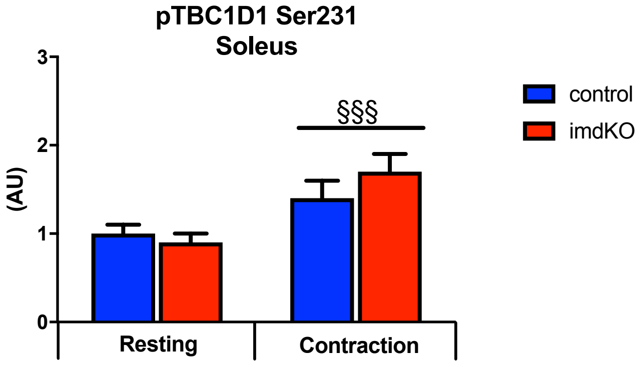

S5J

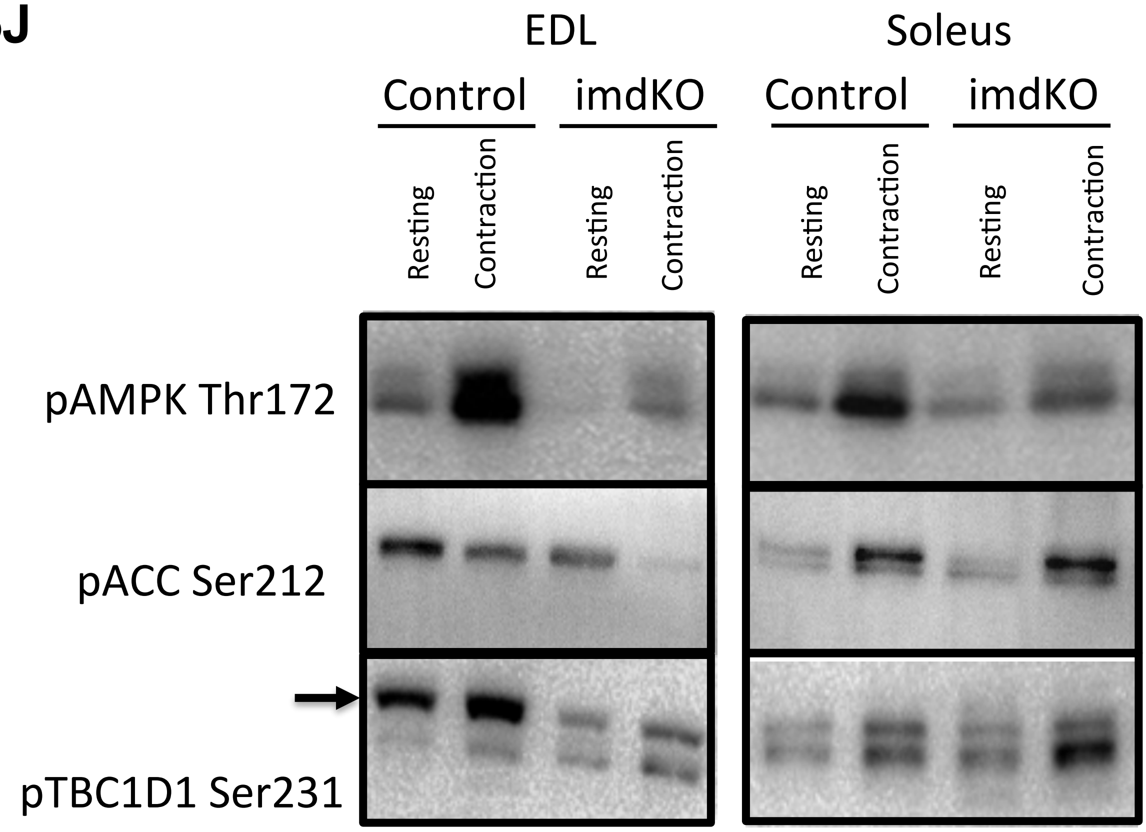

Supplement: Supplementary Figures 3, 4 and 5 [file mmc1.pdf]
