# Supplementary material for: Inducible deletion of skeletal muscle AMPKα reveals that AMPK is required for nucleotide balance but dispensable for muscle glucose uptake and fat oxidation during exercise
Source: Mol Metab. 2020 Jun 3;40:101028. doi: 10.1016/j.molmet.2020.101028 (PMC7356270; doi:10.1016/j.molmet.2020.101028)
Supplement: Supplmental 1: Primer and probe sequences used for real time PCR [file mmc2.docx]

**Supplemental 1**

**Table S1**. Primer and probe sequences used for real time PCR.

| Protein (Gene)  (Primers and probe) | Sequence | Fluorophore/  Quencher |
| --- | --- | --- |
| AMPKγ1 (Prkag1) |  | FAM/TAMRA |
| Forward | 5'-CAAGTTCCAAGTTGGTGGTATTTG-3' |  |
| Reverse | 5'-CGAACACCATTGGTCACCAG-3' |  |
| Probe | 5'-CACTTCGCTACAGGTAAAGAAAGCCTTTTTTGC-3' |  |
|  |  |  |
| AMPKγ3 (Prkag3) |  | FAM/TAMRA |
| Forward | 5'-GCACTGCCTGTGGTCAATGA-3' |  |
| Reverse | 5'-TGCTGGGCAGCCAGGTGAA-3' |  |
| Probe | 5'- CTGGTCAGGTCGTGGGCCTCTACTCC-3' |  |
|  |  |  |
| AMPKα1 (Prkaa1) | Mm01296700_m1 (ThermoFisher Scientific) | FAM/MGB-NFQ |
| AMPKα2 (Prkaa2) | Mm01264789_m1 (ThermoFisher Scientific) | FAM/MGB-NFQ |
| AMPKβ2 (Prkab2) | Mm01257133_m1 (ThermoFisher Scientific) | FAM/MGB-NFQ |
| TATA Box Binding Protein (TBP)  UTP—glucose-1-phosphate uridylyltransferase (Ugp2)  Forward  Reverse  Probe | Mm00446973 _m1 (ThermoFisher Scientific)  5'-CGCAGGATCTCTGACCATGA-3'  5'-AAACCTTCTTAGATAATCTTGAACCTT-3'  5'-CGTGAATTCCCTACAGTGCCCTTGGTT-3' | FAM/MGB-NFQ  FAM/TAMRA |

Primer and probe sequences are given for real time PCR. FAM (5' 6-carboxyfluorescein), TAMRA (5' 6-carboxy-N,N,N',N'-tetramethylrhodamine) and MGB-NFQ (minor groove binder and 3' nonfluorescent quencher).
