# Supplementary material for: Inducible deletion of skeletal muscle AMPKα reveals that AMPK is required for nucleotide balance but dispensable for muscle glucose uptake and fat oxidation during exercise
Source: Mol Metab. 2020 Jun 3;40:101028. doi: 10.1016/j.molmet.2020.101028 (PMC7356270; doi:10.1016/j.molmet.2020.101028)
Supplement: Supplemental 2: List of antibodies [file mmc3.docx]

**Supplemental 2: List of antibodies**

| **Primary Antibodies** | **Product nr.** | **Source** |
| --- | --- | --- |
| pAMPKα Thr172 | #2531 | Cell Signaling Technology, Danvers, MA, USA. |
| AMPKα1 |  | Kindly provided by Prof. Olga Göransson, Lund University. |
| AMPKα2 | #sc-19131 | Santa Cruz Biotechnology, Santa Cruz, CA, USA. |
| AMPKβ2 |  | Kindly donated by Dr. Hardie, Dundee University, UK. |
| AMPK γ1 | #32508 | Abcam, Cambridge, UK. |
| AMPK γ3 | #YZ6229 | Yenzym, San Francisco, CA, USA. |
| FABPpm |  | Kindly donated by Dr. Calles-Escandon, Wake Forest University,Winston-Salem,NC, USA. |
| HKII | #C64G5 | Cell SignalingTechnology, Danvers, MA, USA. |
| GLUT 4 | #PA1-1065 | Thermo Scientific, Waltham, MA, USA. |
| CD36 | #AF2519 | R&D Systems, Minneapolis, MN, USA. |
| GS |  | Kindly donated by Prof. Oluf Pedersen, University of Copenhagen, Copenhagen, DK. |
| ACCβ | #P0397 | Streptadivin–horseradish peroxidase; Dako, Glostrup, Denmark. |
| pACCβ Ser212 | #07-303 | Upstate Bio- technology, Lake Placid, NY, USA. |
| pTBC1D1 Ser231 | #07-2268 | Millipore, Temecula, CA, USA. |
| TBC1D1 |  | Kindly donated by Dr. Hardie, Dundee University. |
| pTBC1D4 Thr642 | #4288 | Cell Signaling Technology; Danvers, MA, USA. |
| TBC1D4 | #07-741 | Millipore, Temecula, CA, USA. |
| Akt2 | #3063 | Cell Signaling Technology; Danvers, MA, USA. |
| pAkt Thr308 | # 9275 | Cell Signaling Technology; Danvers, MA, USA. |
| GAPDH | #2118 | Cell Signaling Technology; Danvers, MA, USA. |
| UGP2 | # Ab157473 | Abcam, Cambridge, UK. |
| OXPHOS AB cocktail | (#ab110413 | Abcam, Cambridge, UK. |
| GP |  | Biogenesis, Poole, UK. |
| UGP2 | # ab157473 | Abcam, Cambridge, UK. |

Secondary antibodies were all horseradish peroxidase–conjugated species-specific immunoglobulins (Jackson ImmunoResearch Laboratories, West Grove, PA, USA; or Dako, Denmark).
