# Supplementary material for: Inducible deletion of skeletal muscle AMPKα reveals that AMPK is required for nucleotide balance but dispensable for muscle glucose uptake and fat oxidation during exercise
Source: Mol Metab. 2020 Jun 3;40:101028. doi: 10.1016/j.molmet.2020.101028 (PMC7356270; doi:10.1016/j.molmet.2020.101028)
Supplement: Supplementary Figure Legends [file mmc4.docx]

**Supplementary Figure Legends**

**Supplemental 3:**

**Figure S3: AMPK protein levels in quadriceps and EDL**

A-E: Protein levels of AMPKα subunits in quadriceps muscle and AMPKβ2, AMPKγ1 and AMPKγ3 in EDL muscle was determined by immunoblotting. Muscle-specific deletion of AMPK α1 and α2 was obtained by expressing a tamoxifen-inducible Cre-recombinase construct driven by the human skeletal muscle actin promotor. All mice received the tamoxifen treatment protocol consisted of three single injections (40 mg/kg bw) each seperated by 48 hours and were investigated 1, 3 and 8 weeks after the last tamoxifen injection. Vehicle-control mice were injected with sunflower seed oil. Data have been normalized to control mice (=100%). Data are given as means ± SEM (n=5-6 within each group). F-G: Representative immunoblotting for AMPK subunits in EDL and heart. H-I: Representative western blotting for protein levels for FABPpm, CD36 and mitochondrial complex proteins in TA muscle. J: WT and AMPKα imdKO were rested or performed 30 min of treadmill exercise and pAMPK Thr172, pACC Ser212 and pTBC1D1 Ser231 (upper band) was investigated in quadriceps muscle by immunoblotting. K: Representative Lineweaver-Burk-plot for calculation of Vmax and Km for AMPD in quadriceps muscle from control and AMPKα imdKO mice.
One-way ANOVA was used for comparing 1 wk, 3 wks and 8 wks to vehicle control within AMPKα imdKO mice. Additional t-test was applied for comparison of AMPKα imdKO with control mice within each given time point. * p≤0.05, ** p≤0.01 and *** p≤0.001 for different from corresponding control mice within time point. (#) p≤0.1, # p≤0.05, ## p≤0.01 and ### p≤0.001 for different from vehicle AMPKα imdKO. † p≤0.05, †† p≤0.05 and ††† p≤0.001 for different from 1 week AMPK α imdKO.

**Supplemental 4:
Figure S4: Substrate utilization, VO_2_ and physical activity in chow fed, HFD or fasted AMPKα imdKO mice.**

A-C: RER in control and AMPKα imdKO mice during the light and dark period in chow and high fat diet fed conditions. D-E: RER in control and AMPKα imdKO subjected to 24 hours of fasting. F-J: VO_2_ in control and AMPKα imdKO during chow, HFD fed and 24 fasted conditions. K-O: Physical activity levels for control and AMPKα imdKO mice measured as beam breaks during chow, HFD fed and 24 fasted conditions. P: Food intake was measured for chow and HFD experiments. Data are presented as means ± SEM (n=8). § p≤0.05, §§ p≤0.01 and §§§ p≤0.001 for effect of dark period compared to light period.

**Supplemental 5:
Figure S5: Force production and cellular signaling in isolated contracted EDL and soleus muscle.**

A-B: Isolated EDL and soleus muscles from control and AMPKα imdKO mice were electrically forced to contract for 10 min while force production was measured (n=4-8). C: Maximal force development during an electrically-induced single twitch. D-I: AMPK Thr172, ACC Ser212 and TBC1D1 Ser231 phosphorylation was investigated in rested and contracted EDL and soleus muscle samples (n=4-8). J: Representative western blotting for pAMPK Thr172, pACC Ser212 and pTBC1D1 Ser231 (upper band) in rested and contracted muscles. Data are given as means ± SEM. Line indicates main effect. * p≤0.05, ** p≤0.01 and *** p≤0.001 effect of genotype. §§ p≤0.01 and §§§ p≤0.001 for effect of intervention/different from resting. Line indicates main effect.
